# Pregnancy and lactation induce distinct immune responses to COVID-19 booster vaccination and SARS-CoV-2 breakthrough infection

**DOI:** 10.1172/jci.insight.191930

**Published:** 2025-07-22

**Authors:** Kailin Yin, Lin Li, Xiaoyu Luo, Jason Neidleman, Arianna G. Cassidy, Yarden Golan, Nida Ozarslan, Christine Y. Lin, Unurzul Jigmeddagva, Mikias Ilala, Megan A. Chidboy, Mary Prahl, Stephanie L. Gaw, Nadia R. Roan

**Affiliations:** 1 Gladstone Institutes, San Francisco, California, USA.; 2 Department of Urology,; 3 Division of Maternal-Fetal Medicine, Department of Obstetrics, Gynecology, and Reproductive Sciences,; 4 Center for Reproductive Sciences, Department of Obstetrics, Gynecology, and Reproductive Sciences,; 5 Department of Bioengineering and Therapeutic Sciences,; 6 Division of Experimental Medicine, Department of Medicine, and; 7 Division of Pediatric Infectious Diseases and Global Health, Department of Pediatrics, UCSF, San Francisco, California, USA.

**Keywords:** Immunology, Reproductive biology, Adaptive immunity, T cells

## Abstract

The widespread uptake of COVID-19 vaccines by women provided a unique opportunity to study the effects of pregnancy and lactation on immune responses to vaccination. Leveraging a cohort with well-defined SARS-CoV-2 exposure history, we found that the magnitude of humoral and cellular immune responses to vaccine-delivered SARS-CoV-2 spike was not affected by pregnancy or lactation status. However, vaccination during pregnancy elicited more stem-like SARS-CoV-2–specific CD4^+^ T cells. Moreover, breakthrough infection promoted spike-specific IgG in pregnant individuals in contrast with IgA in those lactating, suggesting that the pregnancy-to-lactation transition favors mucosal antibody responses. Breakthrough infection also reduced peripheral cytolytic SARS-CoV-2–specific CD8^+^ T cell frequencies during lactation but not pregnancy, which may reflect trafficking of the cells to mammary glands. Our study also uncovered an impact of pregnancy and lactation on global T cell phenotypes. In particular, lactating individuals preferentially exhibited a state of diminished T cell activation. Furthermore, breakthrough infection during pregnancy, but not lactation, diminished frequencies of activated CD8^+^ T cells, tissue-homing CD8^+^ T cells, and γδ T cells. Our findings support the notion that immunity during pregnancy and lactation adapts to benefit the fetus or breastfed infant, with implications for eliciting effective long-term immunity for these uniquely vulnerable groups.

## Abstract 2

While pregnancy and lactation do not affect quantities of vaccine-elicited antibodies and T cells, they affect their features, with implications for mucosal and long-lived immunity.

## Graphical abstract

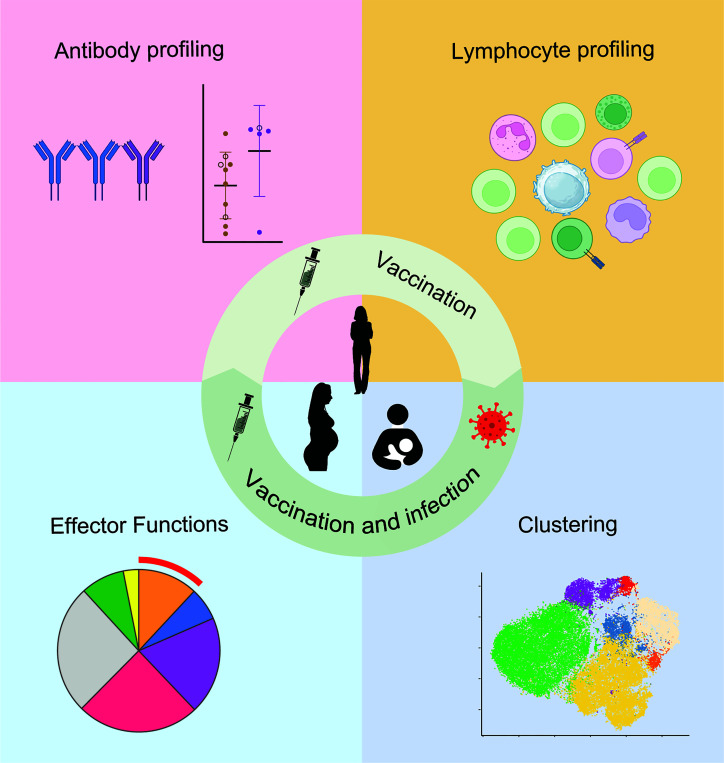

## Introduction

Pregnancy and lactation are accompanied by hormonal changes that can have significant effects on immunity. Pregnancy results in the elevation of estrogen, progesterone, and prolactin levels, and elicits a uniquely tolerant state toward the semiallogeneic fetus and placenta — a phenomenon known as fetomaternal tolerance. This state, however, can diminish maternal immunity and put pregnant people at increased risk of infectious diseases. For example, pregnant individuals exhibit disproportionate morbidity and mortality following malaria, listeria, HIV, influenza, and SARS-CoV-2 infections, among others (1–5).

Whether and how fetomaternal tolerance affects the response to vaccination is less clear, but is important to establish in order to gauge the extent to which effective vaccine-elicited immunity can be generated during this crucial phase of the human life cycle. In mice, pregnancy diminishes T follicular helper (Tfh) and CD8^+^ T cell expansion after de novo exposure to a vaccine antigen in the form of adjuvanted protein (6, 7). Further supporting the notion of potentially suboptimal vaccine responses during pregnancy are studies showing that vaccination with a Zika virus (ZIKV) vaccine elicits a diminished T cell response in pregnant as compared with nonpregnant mice (8). In humans, studies of vaccine immunogenicity during pregnancy have yielded conflicting findings. Most studies have found that flu vaccination elicits similar anti-flu antibody levels in pregnant and nonpregnant people, although some have reported lower titers during pregnancy (4). On the other hand, T cell responses to flu vaccines may be more polyfunctional during pregnancy, with increased CCL4 production (9). An important caveat is that studies of flu vaccination in human cohorts are confounded by the heterogeneous and difficult-to-define prior history of exposure to various vaccines or strains of influenza. As a result, much remains unknown about the immune response to a new antigen via vaccination during the tolerogenic immunologic state of human pregnancy.

The rapidly developed COVID-19 vaccines provided a unique opportunity to fill this knowledge gap. Indeed, the COVID-19 vaccines were the first widely used primary vaccines ever to be given to pregnant individuals — in contrast with vaccines for influenza, pertussis, or respiratory syncytial virus, which were used or trialed on pregnant individuals who had prepregnancy antigen exposure. Although pregnant individuals were excluded from phase III COVID-19 mRNA vaccine trials, large cohort studies from multiple countries have since shown the vaccine to be safe and effective when administered during pregnancy (10), and to date more than 76% of pregnant individuals have received a COVID-19 vaccine (11). Studies have leveraged this situation to compare the response to COVID-19 vaccination in pregnant versus nonpregnant individuals, focusing primarily on antibody responses. Most of these studies found that titers of SARS-CoV-2 antibodies and neutralizing antibodies were similar between vaccinated pregnant and nonpregnant individuals (12–16), although some studies reported lower titers in the context of pregnancy (17, 18). Functional profiles of SARS-CoV-2 vaccine–elicited antibodies were also similar, although antibody effector functions were delayed in pregnant individuals after the first vaccine dose prior to normalizing after the second dose (12). Compared with antibody responses, T cell responses following COVID-19 vaccines have been much less studied in pregnancy. Quantitation of T cell responses by ELISPOT and intracellular cytokine staining for IFN-γ production following peptide stimulation revealed similar responses between pregnant and nonpregnant people (13). However, IFN-γ secretion is just one of many effector functions that T cells can elicit, and hence our overall understanding of the functional and phenotypic features of vaccine-elicited T cells during pregnancy remains rather limited. In fact, in-depth analysis of how pregnancy affects global T cell phenotypes has been limited. Although studies have applied immunophenotyping by flow cytometry to specimens collected during pregnancy, phenotyping was limited to a few markers and/or no comparisons were made to the nonpregnant state (19–21).

Lactation, which immediately follows pregnancy, is characterized by a drop in estrogen and progesterone and elevated levels of prolactin. High prolactin levels during lactation have been associated with increases in CD8^+^ T cell frequencies (22). In addition, breast milk is known to carry maternal IgA and IgG antibodies that are transferred to infants during breastfeeding (22–24) and can protect them against a variety of infectious diseases (21). T cells from breast milk also exhibit distinct phenotypes, TCR repertoires, and transcriptional and functional features relative to their blood counterparts (23, 24). However, overall, knowledge about the impact of lactation on human immune responses is extremely limited, and for the most part has been limited to disease states such as peripartum cardiomyopathy (22), autoimmune diseases (25, 26), and breast cancer (27). A recent study analyzing SARS-CoV-2–specific T cells after COVID-19 vaccination during lactation found expression of a variety of chemokine receptors, but did not include pregnant or nonlactating groups for comparison (23).

Here, we took advantage of the recent large-scale administration of the COVID-19 mRNA vaccines to establish a basic understanding of immune responses upon vaccine antigen exposure during pregnancy and lactation, as compared to the nonpregnant state. We leveraged the COVID-19 Vaccination in Pregnancy and Lactation (COVIPAL) cohort, and analyzed samples collected from 55 individuals who were pregnant, lactating, or neither when they received the third dose of a COVID-19 mRNA vaccine. We also compared vaccine-only versus hybrid immunity by comparing the vaccinated individuals to those who experienced a breakthrough infection after third-dose vaccination. We examined T cells using cytometry by time of flight (CyTOF), a method for in-depth phenotyping of single cells that we have previously implemented in the context of mild and severe COVID-19, vaccination, hybrid immunity, and long COVID in nonpregnant and nonlactating states (28–32). In total, 80 specimens were phenotyped for total and SARS-CoV-2–specific CD4^+^ and CD8^+^ T cells to define the extent to which pregnancy and lactation affects T cell responses to vaccination and hybrid exposure. This was further paired with serological analysis of the same specimens to examine associations between the cellular and humoral responses.

## Results

### Study design.

**Figure 1:**
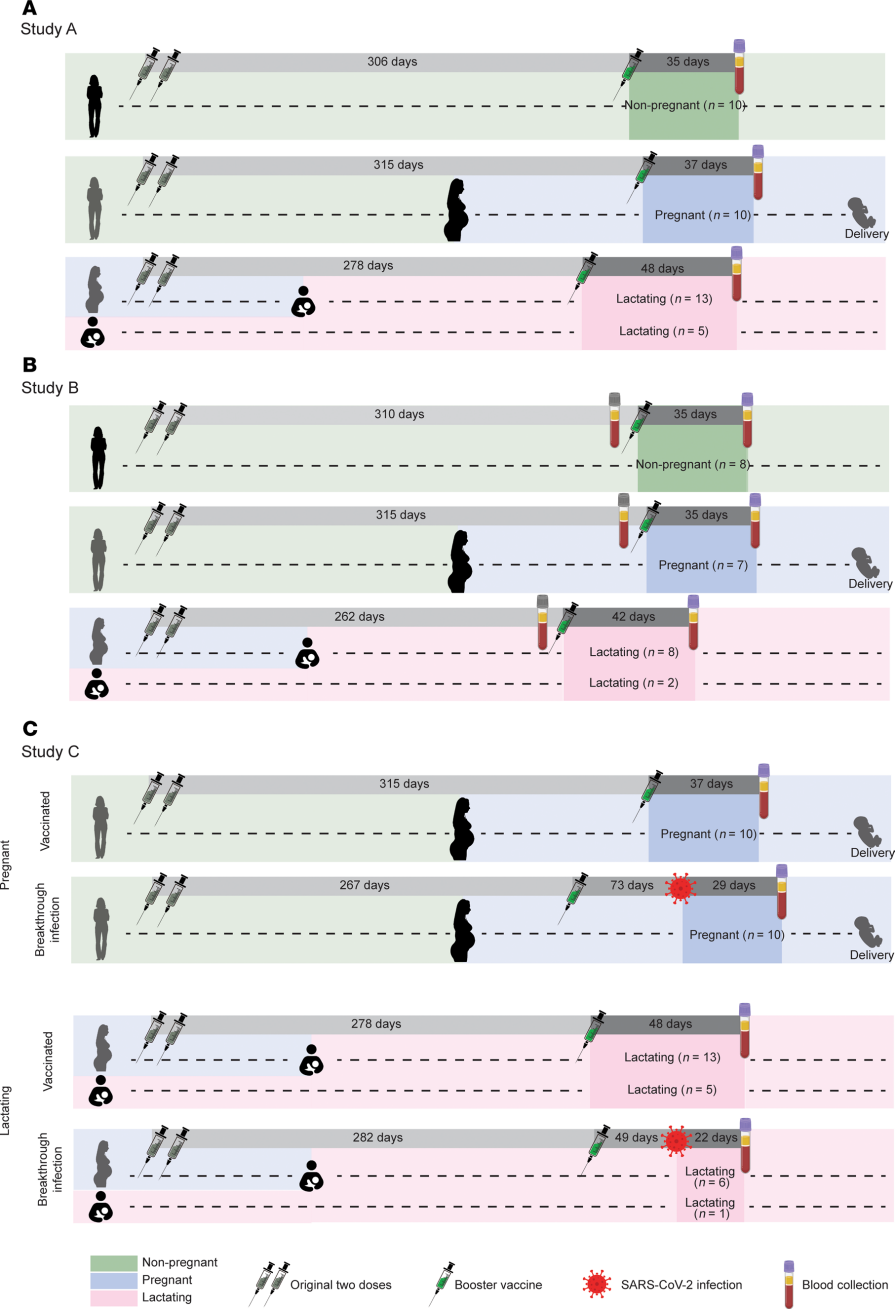
Study design. (**A**) Design for Study A comparing vaccine-elicited immune responses in nonpregnant, pregnant, and lactating individuals. Blood specimens were procured from participants who had received 3 doses of an mRNA COVID-19 vaccine. The nonpregnant group (*n* = 10) consisted of biologically female participants of reproductive age (18–45 years old) not currently pregnant and/or lactating. The pregnant group (*n* = 10) consisted of individuals who were not pregnant at study initiation and pregnant at time of specimen collection. The lactating group (*n* = 18) consisted of 13 individuals who were pregnant at study initiation and lactating at the time of specimen collection, and 5 individuals who were lactating for the duration of the study. (**B**) Study B consisted of longitudinal sampling in a subset of the participants in panel **A** to compare changes in antigen-specific T cell responses in nonpregnant, pregnant, and lactating individuals following boosting. The nonpregnant group (*n* = 8) was comprised of participants not currently pregnant and/or lactating, and the pregnant group (*n* = 7) was comprised of individuals who were not pregnant at study initiation and pregnant during specimen collection. The lactating group (*n* = 10) was comprised of 8 individuals who were pregnant at study initiation and lactating during specimen collection, and 2 individuals who were lactating for the duration of the study. (**C**) Design for Study C comparing breakthrough versus vaccine-only immunity in pregnant and lactating individuals. Blood specimens were procured from pregnant (top) or lactating (bottom) participants who had received 3 doses of an mRNA COVID-19 vaccine with or without subsequent breakthrough infection with SARS-CoV-2. In the vaccinated-only group, pregnant individuals (*n* = 10) were comprised of individuals who were not pregnant at study initiation and pregnant at time of specimen collection. Lactating individuals (*n* = 18) were comprised of 13 individuals who were pregnant at study initiation and lactating at the time of specimen collection, and 5 individuals who were lactating for the duration of the study. In the breakthrough infection group, pregnant individuals (*n* = 10) were comprised of individuals who were not pregnant at study initiation and pregnant at time of specimen collection. Lactating individuals (*n* = 7) were comprised of 6 individuals who were pregnant at study initiation and lactating at the time of specimen collection, and 1 individual who was lactating for the duration of the study. All groups were defined based on the physiological state of the participant at the time of sample collection. Indicated are the timelines of primary vaccination, boosting, breakthrough infection, and blood collection. All numeric values correspond to the mean.

Our overall study design consisted of 3 substudies. Study A consisted of a cross-sectional study design, and consisted of nonpregnant (*n* = 10), pregnant (*n* = 10), and lactating (*n* = 18) individuals enrolled in COVIPAL (Figure 1A and Supplemental Table 1; supplemental material available online with this article; https://doi.org/10.1172/jci.insight.191930DS1). All participants had received 3 doses of either the Moderna mRNA-1273 or Pfizer/BioNTech BNT162b2 vaccine, and were sampled a mean of 41.5 days (range 12–99, SD = 19) after the third dose of vaccination. Nonpregnant participants were neither pregnant nor lactating during either vaccination or sampling. The pregnant group received the first 2 vaccine doses during the preconception period, and the third dose during pregnancy. The lactating group received the third dose during lactation; of these, 13 were pregnant during the first 2 doses and the remaining 5 were lactating throughout the study period (Figure 1A). A subset of Study A’s participants (*n* = 25) was additionally sampled immediately before or a mean of 37.5 days after (range 12–79, SD = 15.8) the third dose of vaccination; these were comprised of nonpregnant (*n* = 8), pregnant (*n* = 7), and lactating (*n* = 10) individuals. These longitudinal specimens were used in Study B (Figure 1B). Finally, we enrolled participants who had experienced a breakthrough infection after receiving the third COVID-19 vaccine dose during pregnancy or lactation, and sampled them a mean of 24.7 days (range 13–33, SD = 7.6) after the infection. These individuals with hybrid immunity were compared to those with vaccine-only immunity that were sampled a mean of 44 days (range 22–99, SD = 20) after the third dose of vaccination, for Study C (Figure 1C). Matched breakthrough infection specimens from nonpregnant/nonlactating individuals were not available, limiting Study C to cross-sectional comparison of breakthrough infection to vaccine-only immunity among pregnant versus lactating individuals.

Plasma from all collected specimens underwent serological analyses to monitor titers of IgG, IgM, and IgA antibodies against SARS-CoV-2 spike receptor-binding domain (RBD) and nucleocapsid (N). Cryopreserved peripheral blood mononuclear cells (PBMCs) from the same time points were analyzed for total and SARS-CoV-2–specific T cell responses by CyTOF. For phenotyping of total T cells, PBMCs were analyzed using a 38-parameter CyTOF phenotyping panel designed to define the subsets, activation and differentiation states, and homing properties of T cells (Supplemental Table 2). Following data acquisition, live, singlet CD4^+^ and CD8^+^ T cells were gated on for further analyses (Supplemental Figure 1A). For identification and characterization of SARS-CoV-2–specific T cells, PBMCs were stimulated for 6 hours in the presence of brefeldin A (BFA) and overlapping 15-mer peptides spanning SARS-CoV-2 ancestral spike, followed by CyTOF analysis. Of the 8 effector molecules in our CyTOF panel, 5 (IFN-γ, TNF, IL-2, IL-21, and CCL4) cleanly identified SARS-CoV-2–specific T cells, defined as cells that only produce these cytokines in the presence of the peptides (Supplemental Figure 1, B and C). Stimulated cells inducing any combination of these cytokines, identified through Boolean gating, were then exported as SARS-CoV-2–specific T cells for further analyses.

### Vaccine-elicited humoral and cellular adaptive immune responses are similar between nonpregnant, pregnant, and lactating individuals.

**Figure 2:**
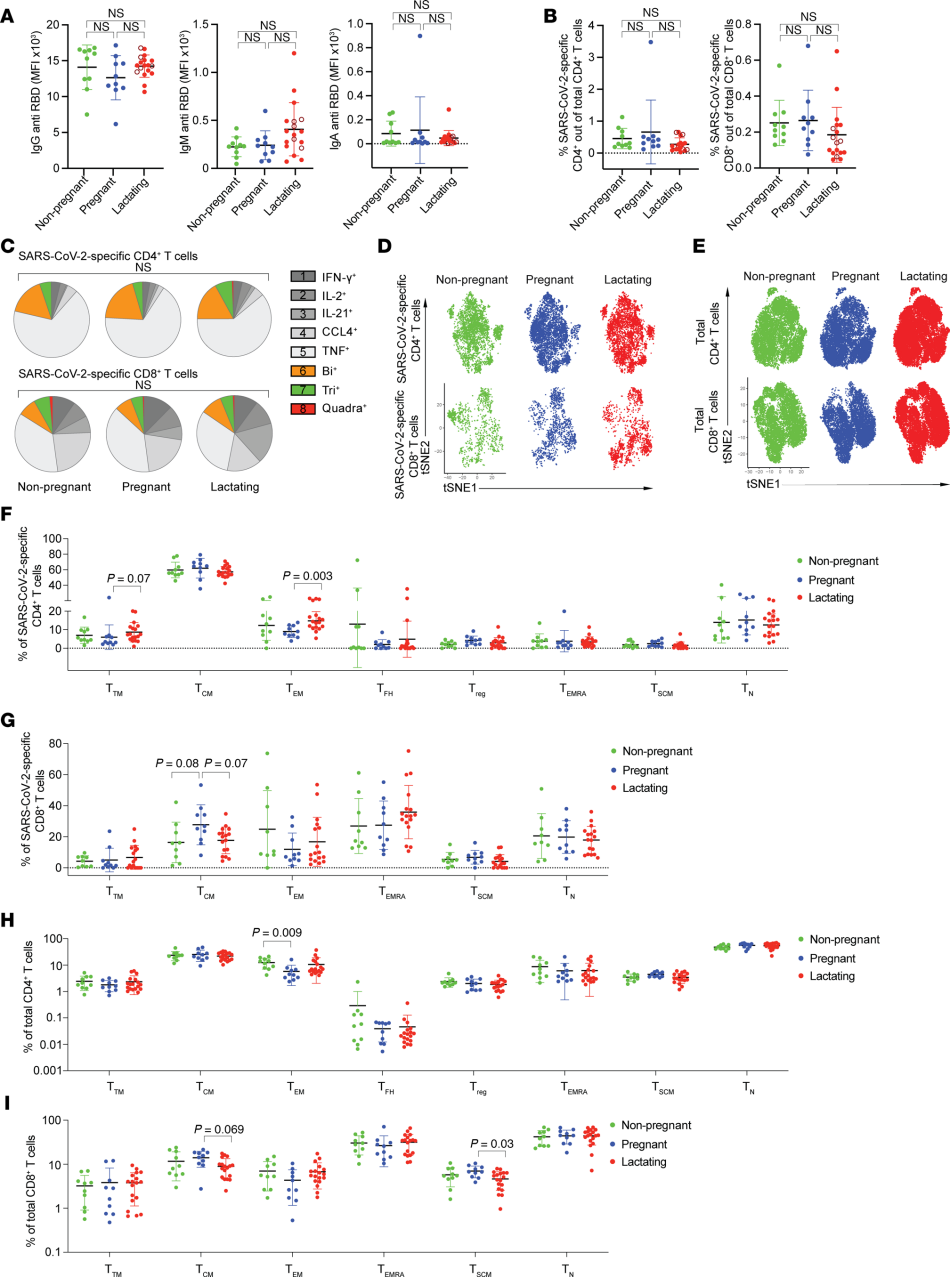
SARS-CoV-2–specific antibody responses and T cell responses are similar between nonpregnant, pregnant, and lactating individuals. (**A**) Dot plots showing relative titers of antibodies against SARS-CoV-2 spike receptor binding domain (RBD) in study participants in Study A as determined by Luminex. Each dot represents 1 participant. Open red circles represent the individuals who were lactating for the duration of the study. (**B**) Dot plots showing percentages of SARS-CoV-2–specific CD4^+^ or CD8^+^ T cells among total CD4^+^ or CD8^+^ T cells, defined as detailed in Methods and Supplemental Figure 1, in participants of Study A. Colors as in panel **A**. NS, nonsignificant as determined by 1-way ANOVA followed by Tukey’s multiple-comparison test. (**C**) SPICE analysis (see Supplemental Methods) showing percentages of polyfunctional (colored) or monofunctional (shades of gray) SARS-CoV-2–specific CD4^+^ (top) and CD8^+^ (bottom) T cells from study participants. NS, nonsignificant as determined by permutation test. (**D** and **E**) t-SNE dot plots depicting SARS-CoV-2–specific (**D**) or total (**E**) CD4^+^ and CD8^+^ T cells from study participants. (**F**–**I**) Dot plots showing percentages of classical T cell subsets among SARS-CoV-2–specific (**F** and **G**) or total (**H** and **I**) CD4^+^ (**F** and **H**) or CD8^+^ (**G** and **I**) T cells from study participants. MFI, median fluorescence intensity; Ttm, transitional memory T cells; Tcm, central memory T cells; Tem, effector memory T cells; Tfh, follicular helper T cells; Treg, regulatory T cells; Temra, CD45RA^+^ effector memory T cells; Tscm, stem cell memory T cells; Tn, naive T cells. Data presented as mean ± SD. *P* values were calculated by 1-way ANOVA followed by Tukey’s multiple-comparison test, Welch’s ANOVA corrected by Dunnett T3 multiple-comparison test, or Kruskal-Wallis *H* test corrected by Dunn’s multiple-comparison test, depending on normality and equality of variance testing. Data from this figure correspond to that generated from *n* = 10 nonpregnant, *n* = 10 pregnant, and *n* = 18 lactating participants.

Serological analysis of Study A (Figure 1A) revealed that RBD-specific IgG, IgM, and IgA titers did not significantly differ between nonpregnant, pregnant, and lactating individuals (Figure 2A). Similarly, there was no difference in the frequency of total SARS-CoV-2–specific T cells (Figure 2B), nor the frequencies of SARS-CoV-2–specific T cells as defined by those individually inducing IFN-γ, TNF, IL-2, IL-21, or CCL4 (Supplemental Figure 2, A and B), between the 3 groups of individuals. The polyfunctionalities of SARS-CoV-2–specific T cells also did not differ between the groups (Figure 2C and Supplemental Figure 2, C and D). Leveraging the full CyTOF panel, we qualitatively assessed for global differences between SARS-CoV-2–specific (Figure 2D) and total (Figure 2E) T cells between the 3 groups, and did not observe drastic differences as assessed by t-distributed stochastic neighbor embedding (t-SNE) visualization of the cells. Accordingly, the distributions of classic CD4^+^ and CD8^+^ T cell subsets (Supplemental Figure 3) were overall similar, except that lactating individuals harbored significantly higher frequencies of SARS-CoV-2–specific CD4^+^ T effector memory (Tem) cells and lower frequencies of total CD8^+^ T stem cell memory (Tscm) cells, as compared with pregnant individuals, while pregnant individuals had lower frequencies of total CD4^+^ Tem cells compared with those who were not pregnant (Figure 2, F–I).

**Figure 3:**
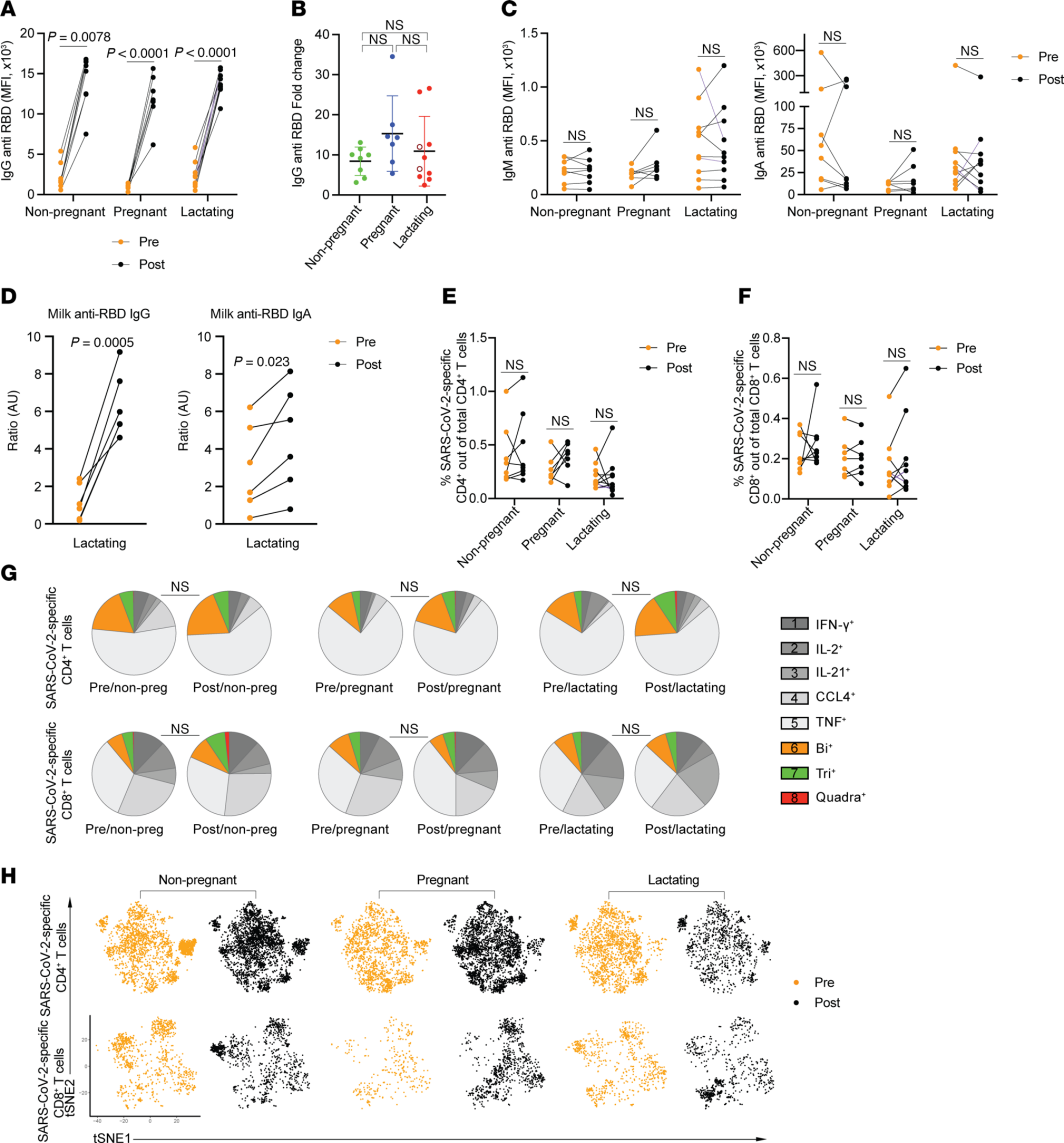
SARS-CoV-2–specific antibody and T cell responses change similarly in nonpregnant, pregnant, and lactating individuals. (**A**) Dot plots showing anti-RBD IgG titers in Study B participants as determined by Luminex. Lines indicate paired samples from the same individuals. (**B**) Dot plots showing fold-changes of anti–SARS-CoV-2 RBD IgG antibody titers in postbooster as compared with prebooster samples among study participants. The individuals who were lactating for the duration of the study are indicated with open red circles. Data presented as mean ± SD. (**C**) Dot plots showing anti-RBD IgM and IgA titers in study participants as determined by Luminex. Lines indicate paired samples from the same individuals. NS, nonsignificant, as determined by 2-sided paired-sample *t* test. MFI, median fluorescence intensity. (**D**) Dot plots showing anti-RBD IgG and IgA titers in milk specimens from study participants as determined by ELISA. Lines indicate paired samples from the same individuals. (**E** and **F**) Dot plots showing percentages of SARS-CoV-2–specific CD4^+^ (**E**) or CD8^+^ (**F**) T cells among total CD4^+^ or CD8^+^ T cells in Study B participants. (**G**) SPICE analysis (see Supplemental Methods) showing frequencies of polyfunctional (colored) or monofunctional (shades of gray) SARS-CoV-2–specific CD4^+^ (top) and CD8^+^ (bottom) T cells from study participants. NS, nonsignificant as determined by permutation test. (**H**) t-SNE dot plots depicting SARS-CoV-2–specific CD4^+^ (top) and CD8^+^ (bottom) T cells from study participants. For all dot plots, *P* values were calculated by 2-sided paired-sample *t* tests, Wilcoxon’s matched-pairs signed-rank test, or Kruskal-Wallis *H* test corrected by Dunn’s multiple-comparison test, depending on normality and equality of variance testing. Data from this figure correspond to that generated from *n* = 8 pregnant, *n* = 7 pregnant, and *n* = 10 lactating participants.

Considering the possibility that the minimal changes observed between the 3 participant groups was due to high intergroup heterogeneity, we next leveraged the longitudinal nature of Study B (Figure 1B) to analyze paired specimens from before versus after the third dose of vaccination. This enabled us to normalize values obtained after booster vaccination to those obtained before. The longitudinal paired blood specimens were subjected to the serological and CyTOF assays implemented for Study A specimens. As expected, the third dose of vaccination significantly boosted anti-RBD IgG titers in all 3 groups (Figure 3, A and B), but not anti-RBD IgM titers (Figure 3C), consistent with this being a recall and not de novo antibody response. Anti-RBD IgA titers were also not boosted by the third vaccination dose (Figure 3C), presumably because the vaccine was administered systemically, resulting in minimal boosting of systemic mucosal IgA responses. However, a subset of our lactating participants was previously analyzed for anti-RBD IgA titers in milk (33), and IgA titers from these participants were boosted by the third vaccination dose, although not as potently as the corresponding IgG titers (Figure 3D). SARS-CoV-2–specific T cell responses also did not differ before and after booster vaccination, irrespective of pregnancy or lactation (Figure 3, E and F). In addition, the polyfunctionality and overall phenotypes of SARS-CoV-2–specific T cells were not altered by the boosting (Figure 3, G and H). Accordingly, the distributions of the classic CD4^+^ and CD8^+^ T cell subsets among SARS-CoV-2–specific T cells were not altered, with the exceptions of SARS-CoV-2–specific CD4^+^ transitional memory T (Ttm) cells and CD8^+^ Tcm cells being increased and CD4^+^ Temra (CD45RA^+^ effector memory T cells) cells being decreased in pregnancy, and SARS-CoV-2–specific CD4^+^ Ttm cells and SARS-CoV-2–specific CD8^+^ Temra cells being increased in lactation, after a third dose of vaccination (Supplemental Figure 4, A–C). Therefore, with the exception of an increase in anti-RBD IgG titers, the third dose of COVID-19 vaccination did not markedly alter adaptive immune responses to SARS-CoV-2. This was observed similarly in nonpregnant, pregnant, and lactating states, suggesting that normalization of the post–third dose data to preboosting conditions still revealed no profound effects of pregnancy or lactation on vaccine-elicited immunity.

### Pregnancy and lactation affect differentiation, homing features, and activation states of total and SARS-CoV-2–specific T cells.

**Figure 4:**
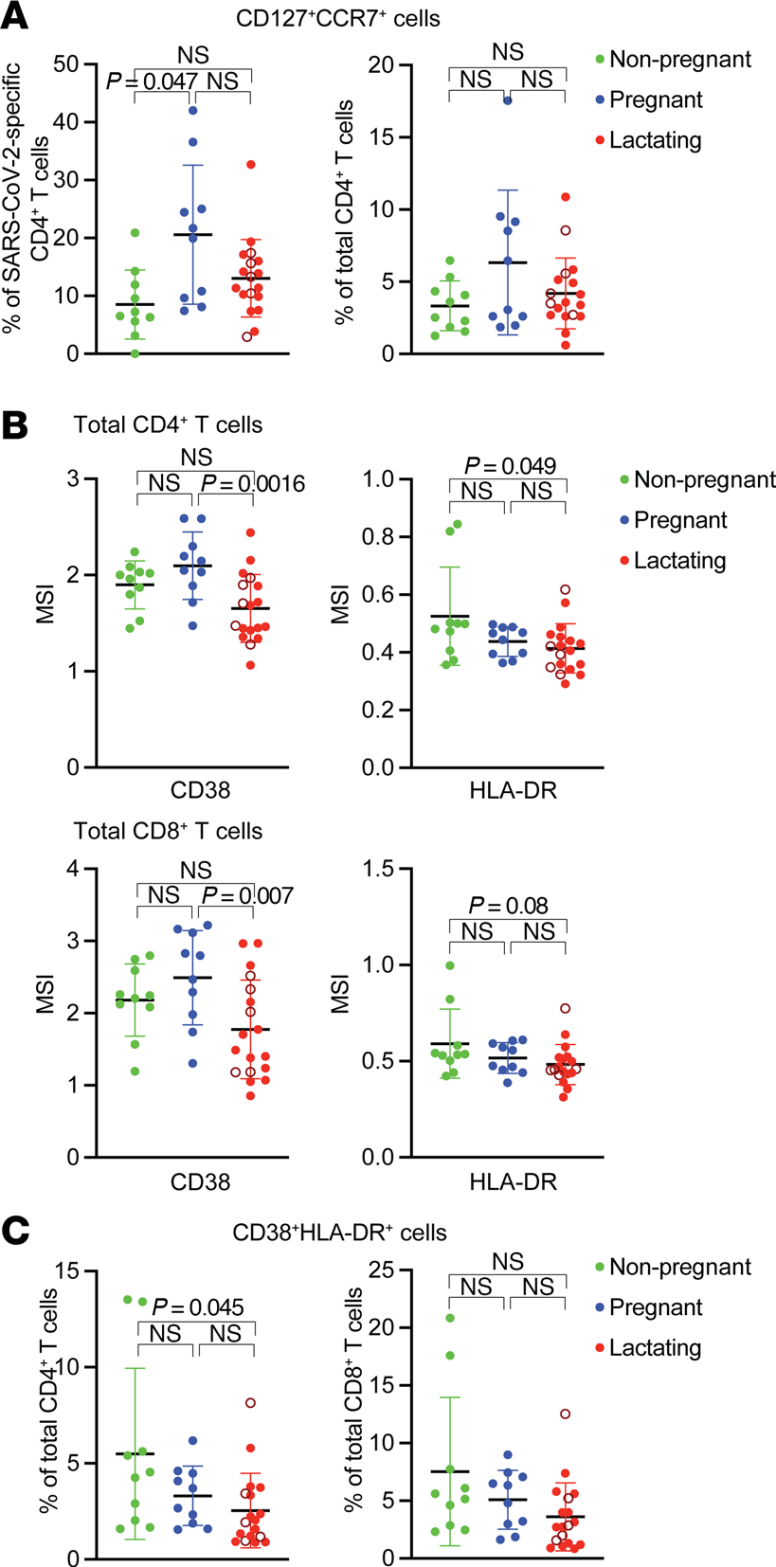
Pregnant individuals exhibit higher proportions of stem-like SARS-CoV-2–specific CD4^+^ T cells, while lactating individuals exhibit overall lower T cell activation. (**A**) Percentages of stem-like CD127^+^CCR7^+^ cells among SARS-CoV-2–specific (left) or total (right) CD4^+^ T cells from participants of Study A. (**B**) Mean signal intensity (MSI) of expression levels of activation markers CD38 and HLA-DR among total CD4^+^ (top) and CD8^+^ (bottom) T cells from study participants. (**C**) Percentages of activated (CD38^+^HLA-DR^+^) cells among total CD4^+^ or CD8^+^ T cells from study participants. In all plots, data are presented as mean ± SD, and dots represent individuals. Open red circles represent the individuals who were lactating for the duration of the study. *P* values were calculated by 1-way ANOVA corrected by Tukey’s multiple-comparison test, or Kruskal-Wallis *H* test corrected by Dunn’s multiple-comparison test, depending on normality and equality of variance testing. Data from this figure correspond to that generated from *n* = 10 nonpregnant, *n* = 10 pregnant, and *n* = 18 lactating participants.

Thus far, our findings suggest that there are no major differences in COVID-19 vaccine responses between nonpregnant, pregnant, and lactating states. To determine whether there were subtle phenotypic differences in the T cells between the 3 groups, we assessed individually the expression levels of each of the 38 phenotyping markers in our CyTOF panel. We first compared the mean expression levels of each of these antigens among SARS-CoV-2–specific CD4^+^ and CD8^+^ T cells, between the nonpregnant, pregnant, and lactating groups in Study A (Supplemental Figure 5). Expression of most antigens did not differ between the groups. However, SARS-CoV-2–specific CD4^+^ T cells from pregnant individuals expressed more CD3, CD4, CD25, and CD27 than did their counterparts from nonpregnant and lactating individuals. Manual gating for select phenotyping markers of interest revealed that SARS-CoV-2–specific CD4^+^ T cells coexpressing the “stem-like” markers CD127 (α chain of the IL-7 receptor involved in homeostatic proliferation) and CCR7 (a marker of long-lived Tcm cells) were significantly increased in pregnant as compared with nonpregnant individuals (Figure 4A). For SARS-CoV-2–specific CD8^+^ T cells, those from nonpregnant individuals expressed higher levels of PD1, and those from pregnant individuals expressed higher levels of CD27, relative to lactating people (Supplemental Figure 5). To further leverage the multidimensional nature of the CyTOF datasets, we performed FlowSOM clustering (34) (see Supplemental Methods). This revealed that the distribution of the clusters of SARS-CoV-2–specific CD4^+^ T cells (clusters A1–A8) and SARS-CoV-2–specific CD8^+^ T cells (clusters B1–B6) did not significantly differ between the 3 participant groups (Supplemental Figure 6, A and B). Likewise, the FlowSOM clusters corresponding to total CD4^+^ T cells (clusters C1–C7) and total CD8^+^ T cells (clusters D1–D7) did not differ in distribution between the groups (Supplemental Figure 6, C and D).

We also leveraged the longitudinal specimens from Study B (Figure 1B) to assess for any phenotypic changes elicited following third-dose COVID-19 vaccination. This revealed interesting patterns of homing receptor expression associated with participant group. In particular, SARS-CoV-2–specific CD4^+^ T cells coexpressing the tissue-homing chemokine receptors CXCR4 and CCR6, or coexpressing CXCR4 together with the integrin component CD29, were significantly decreased by third-dose vaccination in nonpregnant but not pregnant or lactating individuals (Supplemental Figure 7A). Furthermore, SARS-CoV-2–specific CD8^+^ T cells coexpressing chemokine receptors CXCR4 and CCR5 were significantly decreased after third-dose vaccination in nonpregnant but not pregnant or lactating individuals, while those coexpressing CXCR4 and CCR6 were significantly increased in pregnancy but not the other groups (Supplemental Figure 7B).

FlowSOM analysis of the longitudinal specimens revealed no statistically significant changes in cluster distribution after correction for multiple comparisons. However, given that the raw *P* values for some of the comparisons were below 0.05, we as an exploratory analysis assessed for unique features of these clusters that increased or decreased in size after boosting (Supplemental Figure 8, A and B). In lactating individuals only, we observed a decrease (raw *P* = 0.0371) in SARS-CoV-2–specific CD4^+^ T cell cluster A5 (Supplemental Figure 8A), defined by high expression of lymph node homing receptors CCR7 and CD62L, and Ki67, a marker of recent proliferation. By contrast, these cells exhibited low expression of CXCR4, CCR5, CCR6, and CD29, receptors that direct homing to other tissue sites (Supplemental Figure 8C). Boosting of lactating individuals also diminished (raw *P* = 0.0391) the size of SARS-CoV-2–specific CD8^+^ T cell cluster B1 (Supplemental Figure 8B), which was characterized by preferential expression of stemness-associated marker CD127 and conversely low expression of terminal differentiation marker CD57. This cluster of cells also exhibited low expression of checkpoint molecules TIGIT and CTLA4 (Supplemental Figure 8D). By contrast, boosting of lactating individuals uniquely increased (raw *P* = 0.03) the size of SARS-CoV-2–specific CD8^+^ T cell cluster B4 (Supplemental Figure 8B), which was characterized by elevated expression of cytolytic effectors granzyme B (GzmB) and perforin, and the activation markers CD38 and ICOS (Supplemental Figure 8E). Boosting of nonpregnant individuals also uniquely increased (raw *P* = 0.0391) the size of SARS-CoV-2–specific CD8^+^ T cell cluster B6 (Supplemental Figure 8B), which exhibited features of multiple effector functions, including preferential expression of effector cytokine TNF-α, cytolytic effector GzmB, and CD107a, a marker of recent degranulation (Supplemental Figure 8F).

Interestingly, the phenotypic differences we found among SARS-CoV-2–specific T cells between participant groups were not always observed among total T cells. For example, among total CD4^+^ T cells, those coexpressing CD127 and CCR7 were not significantly increased in pregnancy (Figure 4A), and the expression patterns of antigens differentially expressed among SARS-CoV-2–specific T cells (e.g., CD3, CD4, CD25, and CD27) between the participant groups were not different when examining total T cells (Supplemental Figure 7D and Supplemental Figure 9). One exception, however, was that total CD8^+^ T cells coexpressing CXCR4 and CCR6, like their SARS-CoV-2–specific counterparts, were significantly increased in pregnancy compared with the other 2 groups (Supplemental Figure 7, B and D). Total T cells also exhibited other phenotypic differences between participant groups. Lactating individuals overall expressed lower levels of activation markers (CD38 and HLA-DR), among both total CD4^+^ and CD8^+^ T cells (Figure 4B and Supplemental Figure 9). Accordingly, compared with nonpregnant individuals, those lactating harbored significantly lower frequencies of activated CD4^+^ T cells coexpressing CD38 and HLA-DR (Figure 4C). Interestingly, although we did not observe any changes in the frequencies of NK, NKT-like, or γδ T cells between the 3 participant groups, all of these subsets trended toward being less activated in the lactating group as defined by HLA-DR expression (Supplemental Figure 10).

Taken together, these results suggest that although COVID-19 vaccine responses are largely similar between nonpregnant, pregnant, and lactating individuals, the vaccine may preferentially elicit more long-lived, stem-like SARS-CoV-2–specific CD4^+^ T cells when administered during pregnancy. Furthermore, pregnancy status may affect the homing properties of SARS-CoV-2–specific T cells. Finally, our results unveil a global state of diminished activation among CD4^+^ and CD8^+^ T cells, and to a lesser extent among NK and other innate-like cells, during lactation.

### Breakthrough infection during lactation preferentially elicits an IgA RBD-specific recall response and a de novo IgG N-specific response.

**Figure 5:**
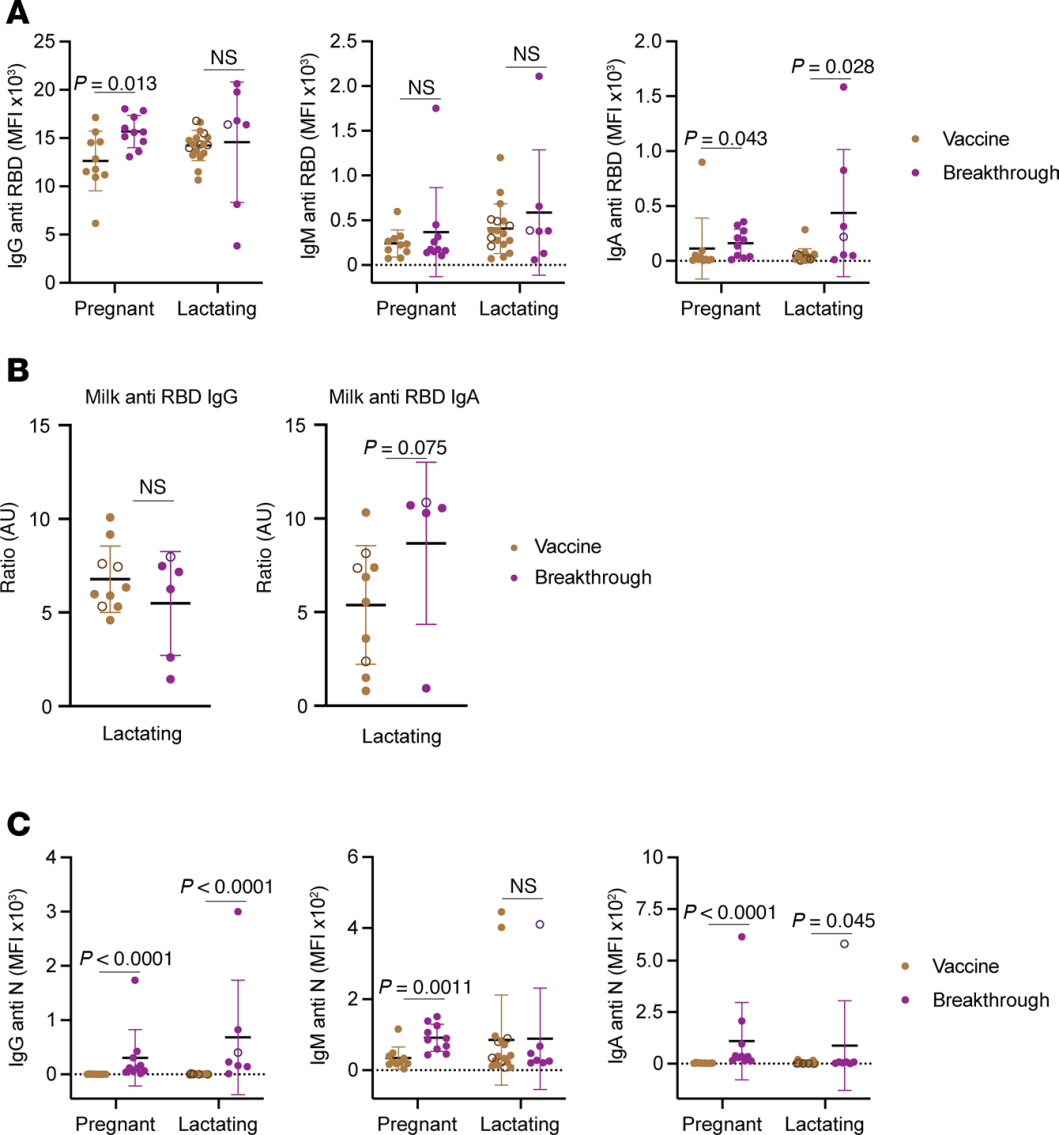
Breakthrough infection differentially boosts SARS-CoV-2–specific antibody isotypes in vaccinated pregnant versus lactating individuals. (**A**) Dot plots showing titers of anti–SARS-CoV-2 RBD IgG, IgM, and IgA in pregnant and lactating participants of Study C. (**B**) Dot plots showing anti-RBD IgG and IgA titers in milk specimens from lactating study participants as determined by ELISA. (**C**) Dot plots showing titers of anti–SARS-CoV-2 nucleocapsid (N) IgG, IgM, and IgA in pregnant and lactating participants of Study C. In all plots, data are presented as mean ± SD, and dots represent individuals. The individuals who were lactating at the time of first 2 vaccine doses (Figure 1C) are indicated with open circles colored brown (vaccination group) and purple (breakthrough infection group). *P* values were calculated by 2-sided Student’s *t* test, Welch’s *t* test, or Mann-Whitney *U* test, depending on normality and equality of variance testing. Data from this figure correspond to that generated from *n* = 10 vaccine and *n* = 10 breakthrough participants in the pregnant group, and *n* = 18 vaccine and *n* = 7 breakthrough participants in the lactating group.

To understand the impact of pregnancy or lactation status on immune responses after breakthrough infection, we leveraged Study C, where participants who had experienced a breakthrough infection after receiving the third COVID-19 vaccine dose during pregnancy or lactation were compared to those who only had 3 vaccine exposures (Figure 1C). Serological analysis of IgG, IgM, and IgA responses against RBD revealed that breakthrough infection stimulated isotype-specific increases in anti-RBD antibody responses in both pregnant and lactating individuals. Breakthrough infection increased both IgG and IgA responses in pregnancy, but only IgA and not IgG responses in lactation (Figure 5A). Analyzing the subset of the lactating participants in Study C that were previously measured for anti-RBD antibody titers in milk (33), we found that there was also a trend for breakthrough infection to induce IgA but not IgG anti-RBD titers in milk (Figure 5B). As expected for a recall response, RBD-specific IgM responses were not increased by breakthrough infection in either group (Figure 5A). These results suggest that lactating people mount a more robust mucosal (IgA) recall antibody response in response to viral respiratory infections than do their pregnant counterparts.

We also quantified N-specific antibody responses as an assessment of de novo antibody responses upon breakthrough infection, since N is not a component of the mRNA-based COVID-19 vaccines and is only present during viral infection. Breakthrough infection significantly increased N-specific IgM titers in pregnant but not lactating individuals. Conversely, it significantly increased N-specific IgG and IgA titers in both pregnant and lactating individuals (Figure 5C). The mechanisms underlying the differential breakthrough infection–induced IgG and IgM responses in pregnant versus lactating individuals are not clear, but could conceivably include altered kinetics, as IgM responses typically peak before IgG responses.

### Breakthrough infection during lactation diminishes cytolytic SARS-CoV-2–specific CD8^+^ T cell frequencies.

As for Studies A and B, we used CyTOF to assess the frequencies and polyfunctionalities of SARS-CoV-2–specific T cells identified by Boolean gating. The frequencies of SARS-CoV-2–specific CD4^+^ and CD8^+^ T cells were not altered by breakthrough infection in either pregnant or lactating groups (Supplemental Figure 11A). The polyfunctionalities of these cells were also not markedly altered, although there was a significant change in the overall functional profile of SARS-CoV-2–specific CD4^+^ T cells in lactating individuals following breakthrough infection (Supplemental Figure 11B). For both groups, the overall phenotypes of the SARS-CoV-2–specific T cells were similar after breakthrough infection (Supplemental Figure 11C), as were classic subset distributions among these cells (Supplemental Figure 11, D and E).

**Figure 6:**
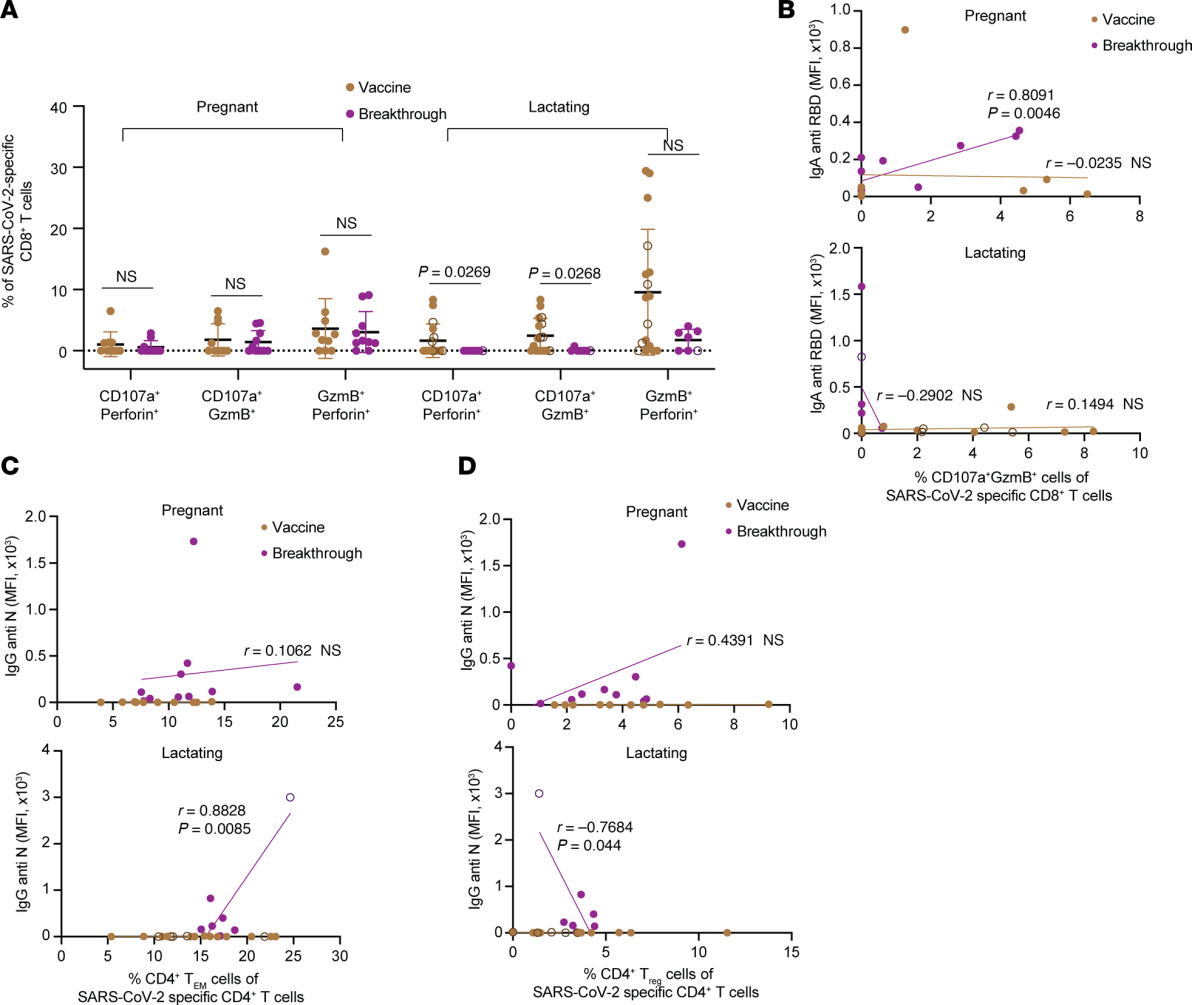
Breakthrough infection diminishes cytolytic SARS-CoV-2–specific CD8^+^ T cell frequencies and coordinates distinct adaptive immune responses in lactating individuals. (**A**) Percentages of CD107a^+^Perforin^+^ cells, CD107a^+^GzmB^+^ cells, and GzmB^+^Perforin^+^ cells among SARS-CoV-2–specific CD8^+^ T cells in pregnant (left) and lactating (right) participants of Study C. *P* values were calculated by Mann-Whitney *U* test or Welch’s *t* test, depending on normality and equality of variance testing. (**B**) The frequencies of cytolytic (CD107a^+^GzmB^+^) SARS-CoV-2–specific CD8^+^ T cells positively associate with anti-RBD IgA titers following breakthrough infection in pregnant but not lactating individuals. (**C**) The frequencies of SARS-CoV-2–specific CD4^+^ Tem cells positively associate with anti-N IgG titers following breakthrough infection in lactating but not pregnant individuals. (**D**) The frequencies of SARS-CoV-2–specific CD4^+^ Treg cells negatively associate with anti-N IgG titers following breakthrough infection in lactating but not pregnant individuals. For all panels, individuals who were lactating for the duration of the study are indicated with open circles colored brown (vaccination group) and purple (breakthrough infection group). For all correlation plots, *r* indicates Pearson’s correlation coefficient, and *P* values were determined by Pearson’s correlation tests. Data from this figure correspond to that generated from *n* = 10 vaccine and *n* = 10 breakthrough participants in the pregnant group, and *n* = 18 vaccine and *n* = 7 breakthrough participants in the lactating group.

FlowSOM analysis revealed no significant differences in cluster distribution within the both the CD4^+^ and CD8^+^ T cell compartments after multiple correction. However, when assessing the raw *P* values, we found that the size of SARS-CoV-2–specific CD8^+^ T cell cluster B6 was smaller in the breakthrough infection group as compared with the vaccine-only group (raw *P* = 0.032), only in lactating individuals (Supplemental Figure 12, A and B). Cluster B6 comprised cytolytic CD8^+^ T cells expressing GzmB and CD107a, a marker of recent granulation. These cells also exhibited low expression of the lymph node homing receptors CD62L and CCR7, as would be expected of cytolytic T cells (Supplemental Figure 12C). The decrease in cluster B6 cells after breakthrough infection in lactating individuals prompted us to investigate whether these individuals exhibited an overall decrease in cytolytic SARS-CoV-2–specific CD8^+^ T cells. Indeed, breakthrough infection during lactation resulted in fewer cytolytic SARS-CoV-2–specific CD8^+^ T cells, and this was not observed during pregnancy. In particular, following breakthrough infection during lactation, SARS-CoV-2–specific CD8^+^ T cells coexpressing the degranulation marker CD107a along with cytolytic effectors perforin or GzmB were significantly decreased; these effects were not observed during pregnancy (Figure 6A).

Interestingly, correlation analyses revealed associations between subsets of SARS-CoV-2–specific T cells with SARS-CoV-2–specific antibody responses that were specific to breakthrough infection cases and that differed between pregnant and lactating groups. For instance, the frequencies of cytolytic SARS-CoV-2–specific CD8^+^ T cells were positively associated with the RBD-specific IgA titer in pregnant but not lactating individuals (Figure 6B), suggesting a connection between mucosal antibody responses and cytolytic T cell responses that is influenced by pregnancy status. In contrast, N-specific IgG titers correlated positively with the frequencies of SARS-CoV-2–specific Tem cells and negatively with the frequencies of SARS-CoV-2–specific CD4^+^ Treg cells in lactation only (Figure 6, C and D). This suggests that effector (Tem) and regulatory (Treg) subsets of SARS-CoV-2–specific memory CD4^+^ T cells may differentially regulate the generation of de novo (N-specific) antibody responses in the context of breakthrough infection, but that the mechanism may be suppressed during pregnancy.

### Breakthrough infection diminishes activated, tissue-homing CD8^+^ T cell frequencies in pregnancy.

**Figure 7:**
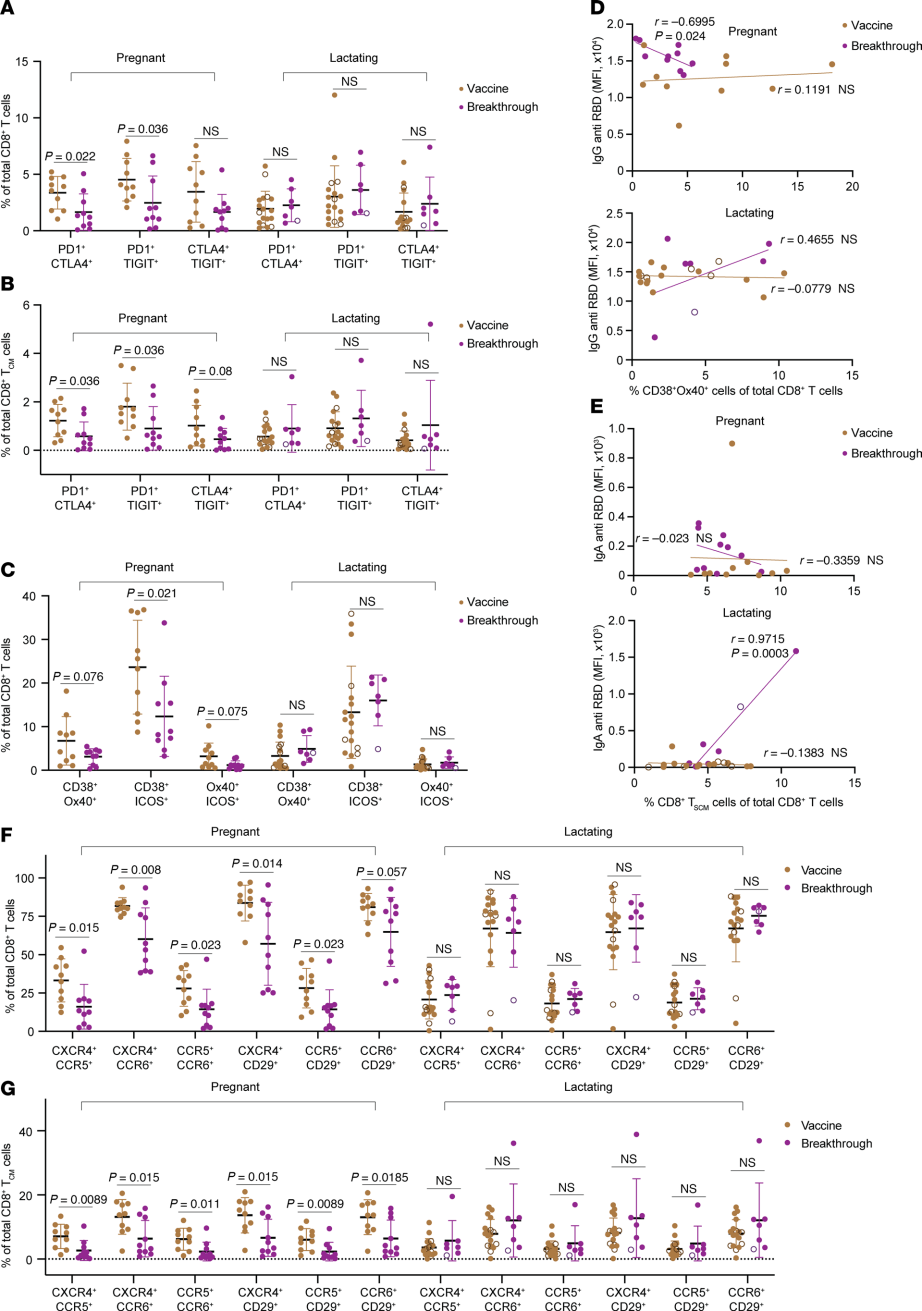
Breakthrough infection diminishes activated, tissue-homing CD8^+^ T cells in pregnant individuals. (**A** and **B**) Percentages of total CD8^+^ T cells coexpressing activation/checkpoint markers PD1, CTLA4, and/or TIGIT among total (**A**) or Tcm (**B**) CD8^+^ T cells of participants from Study C. (**C**) Percentages of total CD8^+^ T cells coexpressing activation markers CD38, Ox40, and ICOS among total CD8^+^ T cells. (**D**) The frequencies of activated (CD38^+^Ox40^+^) CD8^+^ T cells negatively associate with anti-RBD IgG titers following breakthrough infection in pregnant but not lactating individuals. (**E**) The frequencies of CD8^+^ Tscm positively associate with anti-RBD IgA titers after breakthrough infection in lactating but not pregnant individuals. (**F** and **G**) Percentages of total (**F**) or Tcm (**G**) CD8^+^ T cells coexpressing tissue-homing chemokine receptors CXCR4, CCR5, CCR6, and CD29 among total CD8^+^ T cells of study participants. For all panels, individuals who were lactating for the duration of the study are indicated with open circles colored brown (vaccination group) and purple (breakthrough infection group). For all dot plots, *P* values were calculated by 2-sided Student’s *t* test, Welch’s *t* test, or Mann-Whitney *U* test, depending on normality and equality of variance testing. For all correlation plots, *r* indicates Pearson’s correlation coefficient, and *P* values were determined by Pearson’s correlation tests. Data from this figure correspond to that generated from *n* = 10 vaccine and *n* = 10 breakthrough participants in the pregnant group, and *n* = 18 vaccine and *n* = 7 breakthrough participants in the lactating group.

Finally, we assessed for global phenotypic changes among total T cells following breakthrough infection. Although FlowSOM analysis did not reveal differences in cluster distribution between the vaccine-only and breakthrough groups (Supplemental Figure 12B), manual gating revealed that breakthrough infection during pregnancy but not lactation elicited a variety of perturbations to the CD8^+^ T cell compartment. In particular, CD8^+^ T cells coexpressing the checkpoint molecules PD1, CTLA4, and/or TIGIT were diminished by breakthrough infection in pregnant individuals (Figure 7A), and this effect was primarily driven by the Tcm subset of CD8^+^ T cells (Figure 7B). As PD1, CTLA4, and TIGIT also function as activation markers, we assessed the expression levels of other T cell activation markers among total CD8^+^ T cells. This revealed a decrease in CD8^+^ T cells coexpressing various combinations of activation markers CD38, Ox40, and ICOS following breakthrough infection, but again only in the context of pregnancy (Figure 7C). The negative association of activated CD38^+^Ox40^+^ among total CD8^+^ T cells with RBD-specific IgG levels in pregnant but not lactating individuals (Figure 7D) suggests that the general repression of CD8^+^ T cell activation in pregnant patients that experienced breakthrough infection may negatively impact the recall antibody response against SARS-CoV-2. In contrast, lactating mothers, who had a more robust mucosal antibody response to breakthrough infection (Figure 5A), exhibited RBD-specific IgA antibody responses that positively correlated with total CD8^+^ Tscm frequencies (Figure 7E), implicating a role for CD8^+^ Tscm cells in promoting mucosal antibody responses.

In addition to exhibiting a diminished activation state, total CD8^+^ T cells from pregnant individuals that experienced breakthrough infection also expressed lower levels of tissue-homing receptors (CXCR4, CCR5, CCR6, and CD29) (Figure 7F); this effect was again primarily attributable to CD8^+^ Tcm cells (Figure 7G). Finally, analysis of innate-like immune subsets following breakthrough infection revealed no changes among NK or NKT-like in both groups, but a significant decrease in the frequencies of γδ T cells in pregnancy but not lactation (Supplemental Figure 13).

Taken together, these results suggest that breakthrough infection preferentially elicits an overall attenuated state in pregnant (but not lactating) individuals, characterized by diminished expression of activation and homing markers on CD8^+^ T cells, and diminished frequencies of γδ T cells.

## Discussion

Understanding the features of adaptive immunity during pregnancy and lactation is key to understanding reproductive biology and immune tolerance. While immunity during pregnancy is under active investigation, the immunological landscape of lactation is poorly defined. Pregnancy and lactation are distinct states supporting the growing fetus and/or infant nutrition that can impact immunity (35–37). Here, we leveraged the fact that the release of the novel mRNA vaccines during the COVID-19 pandemic provided a first-of-its kind opportunity to characterize antigen-specific responses with clearly defined first and subsequent antigen exposures during pregnancy and lactation.

Overall, we found that the magnitude of humoral and cellular immune responses to COVID-19 vaccination was not affected by pregnancy nor lactation status. Vaccine-elicited SARS-CoV-2 IgG, IgM, and IgA antibody titers were not affected by pregnancy, consistent with prior studies (12, 13), and we further demonstrated that lactational state does not negatively impact serologic responses to COVID-19 vaccines. Studies assessing T cell responses elicited by COVID-19 vaccines in pregnant individuals have been more limited. To our knowledge, only one such study had been conducted prior to ours. That study defined SARS-CoV-2–specific T cells using just a single effector cytokine (IFN-γ) and found no difference in pregnant versus nonpregnant individuals (13). We confirmed that the numbers of vaccine-elicited IFN-γ^+^ SARS-CoV-2–specific T cells did not differ between pregnant and nonpregnant groups, but also monitored 4 additional effector cytokines (TNF, IL-2, IL-21, and CCL4) and 3 cytolytic markers (GzmB, perforin, and CD107a) among SARS-CoV-2–specific T cells. This more detailed assessment of antigen-specific T cell responses also found no differences between pregnant and nonpregnant individuals whether the cells were defined by effector molecule expression individually or in combinations (including assessment of polyfunctionality). Similarly, the numbers and polyfunctionalities of SARS-CoV-2–specific T cells were similar in lactation.

The impact of pregnancy and lactation on the immune response to vaccination only became discernible when we examined the phenotypes and differentiation states of SARS-CoV-2–specific T cells. While the distribution of classic subsets among SARS-CoV-2–specific T cells was for the most part similar between the 3 groups, pregnant individuals harbored lower frequencies of SARS-CoV-2–specific CD4^+^ Tem cells and higher frequencies of total CD8^+^ Tscm cells relative to lactating individuals. In addition, pregnant individuals harbored higher frequencies of CCR7^+^CD127^+^ SARS-CoV-2–specific CD4^+^ T cells, supporting preferential differentiation of antigen-specific T cells with stem-like phenotypes (CCR7 plays role in lymphoid homing and is a marker of long-lived Tcm cells, and CD127 is a key receptor mediating IL-7–driven homeostatic proliferation and is expressed on Tscm cells) (38). Interestingly, a recent study suggested CMV-mediated priming of long-lived CD127^+^ CMV-specific CD4^+^ T cells during pregnancy, and a steady increase in frequencies of these cells over time in a manner that continued after pregnancy (39). Hence, pregnancy may generally facilitate priming of stem-like T cells, in the context of both vaccination and infection.

Consistent with our cross-sectional analysis, our longitudinal analyses (Study B) revealed no difference between the 3 groups in changes in frequencies of SARS-CoV-2–specific T cells before versus after the third dose of COVID-19 vaccination. In fact, in all 3 groups, the frequencies of SARS-CoV-2–specific T cells were the same before versus after third-dose vaccination. This may be due to a plateauing out of T cell responses after the second dose of vaccination, as has been observed in nonpregnant individuals (31, 40–43). In contrast, IgG titers against SARS-CoV-2 were significantly boosted by third-dose vaccination irrespective of pregnancy or lactation status. IgM titers were not boosted, consistent with IgM reflecting de novo responses. Systemic IgA titers were also not boosted by third-dose vaccination, consistent with what has been reported in nonpregnant individuals (33, 44). As IgA is an antibody isotype associated with mucosal responses, the lack of IgA boosting may be due to relatively poor elicitation of mucosal immunity by the current subcutaneously administered COVID-19 vaccines.

By contrast, we found that breakthrough infection induced a robust rise in SARS-CoV-2–specific IgA titers, whereas in pregnant individuals breakthrough infection boosted both IgG and IgA titers. Such diversion of the antibody response from IgG to IgA during the pregnancy-to-lactation transition may serve to benefit the infant, as IgA is the predominant antibody transferred to the infant through breast milk (33) and is breast milk’s main source of SARS-CoV-2 neutralizing antibodies (45). As increased IgA production in breast milk is also associated with protection of breastfed infants against other viruses (46, 47), the preferential elicitation of IgA responses during lactation likely extends beyond COVID-19 immunity. The mechanisms underlying preferential IgA responses during lactation remain to be determined, but interestingly, we observed that SARS-CoV-2–specific IgA titers significantly associated with increased frequencies of total CD8^+^ Tscm cells in lactating people. A link between IgA and Tscm responses has not, to our knowledge, been reported, but as Tscm cells have been linked to favorable long-term durability of COVID-19 and yellow fever vaccine responses (48, 49), it is conceivable that persisting Tscm cells may somehow facilitate crosstalk between the cellular and humoral arms of immunity to promote mucosal immune responses. In that regard, future studies to establish a better understanding of the upstream mediators leading to superior IgA antibody responses in lactating individuals — and exploring the role of Tscm in this process — are warranted, since such knowledge could conceivably be tested in future vaccination strategies to elicit superior mucosal antibody responses in nonlactating individuals.

Although breakthrough infection during lactation preferentially increased SARS-CoV-2–specific IgA production, cytolytic SARS-CoV-2–specific CD8^+^ T cell frequencies were diminished. The mechanisms underlying this diminished cytolytic response during lactation are unknown, but could conceivably reflect preferential trafficking of these cytolytic cells from maternal peripheral circulation to mammary glands. Indeed, a recent study reported elevated expression of cytolytic effectors, including GzmB, among CD8^+^ T cells from breast milk as compared with blood (24). Further consistent with trafficking is the observation that SARS-CoV-2–specific T cells are more frequent in breast milk than in blood after vaccination (23). We also found lactating individuals to harbor fewer activated T cells in blood, relative to their nonpregnant and pregnant counterparts. This could conceivably result from a general trafficking of activated T cells to the mammary glands during lactation. Re-trafficking of T cells to mammary glands can benefit the breastfed infant since immune cells can be directly transferred through breastfeeding through a process known as maternal lactational microchimerism. Interestingly, a mouse study found that CD8^+^ T cells with elevated cytolytic activity were transferred from breast milk to Peyer’s patches of the suckling pups’ intestines, and these cells were postulated to exert protective immunity for the pups (50). Future studies interrogating the protective effects of breast milk–derived T cells for the infant, as well as whether re-trafficking of T cells to the mammary gland occurs and if so how this affects overall immunity for the lactating individual, are warranted.

Breakthrough infection also affected T cell phenotypes of pregnant individuals in unique ways. For instance, it lowered expression of checkpoint/activation molecules PD1, CTLA4, and TIGIT, as well as other activation markers (CD38, ICOS, Ox40) on total CD8^+^ T cells, underscoring a general suppression of CD8^+^ T cell activation. The diminished frequencies of activated T cells may be caused by a re-trafficking of these cells from circulation into tissues, particularly the placenta. Indeed, increased T cell infiltrates are observed in the maternal decidua weeks to months following SARS-CoV-2 infection in pregnant individuals (51). It is also worth noting our finding that pregnant (but not lactating) participants exhibited a decrease in peripheral γδ T cell frequencies following breakthrough infection. γδ T cells are semi-innate cells that play a role in early immune responses, and undergo lymphopenia during acute COVID-19 particularly during severe cases (52, 53). The extent to which diminished frequencies of activated T cells and γδ T cells in circulation during pregnancy affects immunity to SARS-CoV-2 is not clear, but given the increased risk of pregnant individuals for severe COVID-19 (5), it is possible that this systemic attenuation of these T cell populations can have negative effects.

Our study has limitations. The study included a small sample size of only 55 participants with biological variability, and we were not able to recruit matched nonlactating, nonpregnant women for Study C, and hence the effects of breakthrough infection could only be compared between lactating and pregnant women. Furthermore, although all major conclusions were drawn based on statistically significant (*P* < 0.05) results, sometimes the actual fold-changes of the differences — particularly when it came to expression levels of some phenotypic markers or subset frequencies — were not large in magnitude. In addition, although Treg cells are known to play important immunosuppressive roles, the extent to which these cells modulate vaccine responses in pregnancy and lactation was not explored in the current study, but should be explored in future research. Future studies exploring the mechanistic basis of the observations reported herein should also be pursued.

In conclusion, we found that cellular and humoral responses to COVID-19 vaccination are both as robust in pregnant and lactating people as in nonpregnant populations. However, our in-depth phenotyping of immune cells using CyTOF uncovered interesting differences between these different physiological states. Notably, we found a potential distinct advantage of vaccination during pregnancy when stem-like vaccine-specific T cells were preferentially boosted. Conversely, lactation appears to favor elicitation of mucosal immunity in the form of enhanced IgA production following breakthrough infection. At the same time, however, breakthrough infection during lactation results in a marked diminution of systemic levels of cytolytic SARS-CoV-2–specific CD8^+^ T cells, which could put the lactating individual at a disadvantage in clearing SARS-CoV-2 infection. The mechanisms underlying such changes in antigen-specific immune responses during pregnancy and lactation remain to be ascertained, and may be influenced by pregnancy-associated hormones, given that estrogen, progesterone, prolactin, and placental lactogen are all elevated during pregnancy, and prolactin remains elevated during lactation (54, 55), and receptors for these hormones are expressed by a variety of immune cells, including T cells (56–58). Overall, our results suggest that tailoring vaccine strategies to pregnant and lactating individuals — for example by exploring adjuvant-based approaches to increase IgA responses during pregnancy (59) and to enhance boosting of cytolytic T cell responses during lactation — can potentially improve the long-term effectiveness of vaccine-elicited immunity for these uniquely vulnerable groups of individuals.

## Methods

### Sex as a biological variable.

All specimens analyzed in this study were procured from participants assigned female at birth. Individuals that were assigned male at birth were not analyzed in this study, as this study focused on assessing vaccine-elicited immune responses during pregnancy and lactation.

### Study design.

All participants received either the Moderna mRNA-1273 or Pfizer/BioNTech BNT162b2 vaccines. Blood specimens were collected between April 2021 and February 2022 in the San Francisco Bay Area. All participants enrolled in this study were female and of reproductive age. At each study visit, all pregnant or lactating participants were surveyed using a questionnaire inquiring about their pregnancy or lactation status, COVID-19 vaccination status, and whether they had a prior COVID-19 infection. The vaccination group (*n* = 38) had a median age of 37 years and was composed of *n* = 10 nonpregnant, *n* = 18 lactating, and *n* = 10 pregnant participants. Participants in the vaccination group had no history of SARS-CoV-2 infections reported, confirmed by serologic testing demonstrating lack of anti-N antibodies using the antibody assay described further below, and confirmation of no COVID-19 infection by clinical survey at each follow-up study visit. The breakthrough infection group (*n* = 17) had a median age of 36 years and was composed of *n* = 7 lactating and *n* = 10 pregnant participants. All individuals in the breakthrough infection group had their SARS-CoV-2 infections confirmed by a positive PCR test.

### Sample collection.

Peripheral blood was collected in EDTA-containing tubes. PBMCs were isolated by density-gradient sedimentation using Ficoll-Paque (Cytiva). Briefly, cellular fractions were diluted 1:2 with PBS (Corning) and gently layered over 12.5 mL Ficoll in a conical tube, followed by centrifugation for 30 minutes at 800*g* without brake. The PBMC buffy coat in the second layer was then collected and washed with PBS 3 times. Cells were counted, and the PBMCs were aliquoted and cryopreserved in cell freezing media containing 90% heat-inactivated fetal bovine serum (FBS; Sigma-Aldrich) and 10% dimethyl sulfoxide (DMSO; Sigma-Aldrich). The aliquots were placed in Mr. Frosty freezing containers (Thermo Fisher Scientific) at –80°C overnight, and then transferred to liquid nitrogen for storage until processing for CyTOF. Plasma was collected from whole blood as described previously (33) and was immediately cryopreserved at –80°C until analysis. Maternal blood samples in the prevaccination group were collected within 24 hours before vaccination.

### Antibody assays.

IgG, IgM, and IgA antibodies against the spike receptor RBD or N of SARS-CoV-2 were analyzed with a multiplex-based human serology kit (Bio-Rad, 12014777) according to the manufacturer’s instructions. Briefly, plasma samples were diluted at 1:1000 for IgG detection, and 1:100 for IgM and IgA detection. For IgM measurements, samples were mixed with GullSORB IgG inactivation reagent (Meridian Bioscience) at 1:10 to reduce IgG interference prior to diluting. Diluted samples were incubated with RBD or N protein–conjugated beads for 30 minutes at room temperature. Secondary antibodies for IgG, IgM, and IgA were added into respective wells and incubated for 30 minutes at room temperature, followed by a 10-minute incubation with streptavidin-phycoerythrin. After washing and resuspension of the beads, reactions were read out on a BioPlex 200 (Bio-Rad). Results were expressed as median fluorescence intensity (MFI) per 100 beads. Cutoffs were determined using a similar criterion as previously reported (14, 15, 60, 61). MFI less than 10 was considered negative. Positive cutoffs for MFI were 75 and 100 for IgG anti-RBD and anti-N, 250 and 600 for IgM anti-RBD and anti-N, and 80 and 100 for IgA anti-RBD and anti-N. A subset of the lactating participants analyzed in this study were previously analyzed for milk titers of IgG and IgA anti-RBD (33). The milk antibody datasets from the overlapping participants were re-analyzed in the current study.

### Peptide stimulation.

PBMCs were cultured overnight in RPMI 1640 (Corning, 15-040-CV) containing 10% FBS (VWR, 89510-186), 1% L-glutamine (Corning, 25-005-CI), and 1% penicillin-streptomycin (Corning, 30-002-CI) after cell revival. Cells were then treated at 37°C for 6 hours with 0.1% DMSO and 3 μg/mL BFA (eBioscience) as a negative control, or with 3 μg/mL BFA, 2 μg/mL anti-CD28 (catalog 340975, clone L293), and 1 μg/mL anti-CD49d (catalog 340976, clone L25) as a source of costimulation (both from BD Biosciences), and 300 nM overlapping 15-mer SARS-CoV-2 spike peptides (PM-WCPV-S-1, JPT), following a standard intracellular cytokine staining protocol previously implemented (32). ^151^Eu-labeled anti–human CD107a antibody (Standard BioTools, 1 μL per 3 million cells) was added to all samples during the incubation to monitor degranulation. Following the incubation, cells were washed twice with PBS (Rockland, MB-008) containing 0.1% bovine serum albumin (Sigma-Aldrich, A7284) and 0.1% sodium azide (Sigma-Aldrich, S2002), and then fixed for CyTOF analyses as detailed below.

### CyTOF sample fixation and staining.

Six million cells were stained with cisplatin (Sigma-Aldrich) as a live/dead distinguisher and then fixed in 2% paraformaldehyde (Electron Microscopy Science) as described previously (28–32). Cells were stained with Cell-ID 20-Plex barcoding kit (Standard BioTools, 201060), and then with cell surface followed by intracellular antibodies (Supplemental Table 2). Then, Cell-ID Intercalator-Ir–125 µM (Standard BioTools, 201192A) was added according to the Maxpar Nuclear Antigen Staining with Fresh Fix Protocol (Standard BioTools, 400276 A3). After washing with Maxpar Cell Staining Buffer (Standard BioTools, 201068) and Maxpar Cell Acquisition Solution (Standard BioTools, 201240), 0.1× EQ Four Element Calibration Beads (Standard BioTools, 201078) were spiked into the samples. The samples were then passed through using a 35 μm nylon filter (Falcon, 352235) and then loaded on a Helios CyTOF machine (UCSF Parnassus Flow Cytometry Core) for data acquisition.

Further details on CyTOF data analysis, including on-data normalization, identification of subsets of interest, t-SNE visualization, polyfunctionality analysis, and FlowSOM analysis, are available in the Supplemental Methods.

### Statistics.

For statistical analyses, we first identified outliers in any group (*n* > 10) using the robust outlier identification and testing (ROUT) method and then removed them from tests for normal distribution and equality of variance. For comparisons between 3 or more groups, we first performed a normal distribution test using the Shapiro-Wilk test, followed by a test for equality of variance using the Brown-Forsythe test. In situations where both the normality and equality of variance tests passed, we used 1-way ANOVA followed by Tukey’s multiple-comparison correction (parametric test). In situations where the equality of variance test failed but the normality test passed, we used a Welch’s ANOVA test followed by Dunnett T3 multiple-comparison correction (parametric test). In situations where the normality test failed, we used a Kruskal-Wallis *H* test followed by Dunn’s multiple-comparison correction (nonparametric test). For comparisons between 2 unpaired groups, we first performed a normal distribution test using the Shapiro-Wilk test followed by a test for equality of variance using the *F* test. In situations where both the normality and equality of variance tests passed, we used the Student’s *t* test (parametric). In situations where the equality of variance test failed but the normality test passed, we used the Welch’s *t* test (parametric test). In situations where the normality test failed, we used the Mann-Whitney *U* test (nonparametric). For comparisons between paired groups, we performed a normal distribution test using the Shapiro-Wilk test. In situations where the normality test passed, we used a paired-sample *t* test (parametric). In situations where the normality test failed, we used Wilcoxon’s matched-pairs signed-rank test (nonparametric). Significances (adjusted *P* < 0.05) as well as trends (adjusted *P* < 0.1) are reported as actual *P* values, and nonsignificant was defined as anything with a *P* value of greater than 0.1. Data are presented as mean ± standard deviation (SD). Graphs were plotted using GraphPad Prism (version 10.2.0).

### Study approval.

This study was approved by the institutional review board of UCSF (IRB 20-32077 and IRB 19-29713) and written informed consent was obtained from all study participants.

### Data availability.

The raw CyTOF and serology data for this study are accessible via the following link: https://datadryad.org/dataset/doi:10.5061/dryad.gb5mkkwxh

All data values associated with the main manuscript and supplemental material are summarized in the Supporting Data Values file.

## Author contributions

KY designed the experiments, performed CyTOF, conducted data analyses, and prepared the figures and tables. LL designed the experiments, performed antibody assays, conducted data analysis, and prepared figures and tables. XL designed pipelines for CyTOF data analyses. JN prepared peptides and consulted on experiments. AGC designed the pregnancy cohort and recruited participants. YG designed the lactation cohort, recruited participants, and designed and conducted breast milk antibody experiments. NO prepared patient samples for experiments and assisted with experiments. CYL recruited participants and prepared patient samples for experiments. UJ prepared patient samples for experiments and assisted with experiments. MI prepared patient samples for experiments and assisted with breast milk antibody experiments. MAC prepared patient samples for experiments and consulted on experiments. MP designed the patient cohorts, performed supervision, and conducted data analysis and interpretation. SLG designed the patient cohorts, performed supervision, conducted data analysis and interpretation, and prepared figures and tables. NRR oversaw the study, performed supervision, conducted data analyses, prepared figures and tables, and wrote the first draft of the manuscript. All authors have read and approved this manuscript.

## Supplementary Material

Supplemental data

Supporting data values

## Version 1 07/22/2025

Electronic publication
