# Supplementary material for: Pregnancy and lactation induce distinct immune responses to COVID-19 booster vaccination and SARS-CoV-2 breakthrough infection
Source: JCI Insight. 2025 Jul 22;10(14):e191930. doi: 10.1172/jci.insight.191930 (PMC12288967; doi:10.1172/jci.insight.191930)
Supplement: Supplemental data [file jciinsight-10-191930-s122.pdf]

SUPPLEMENTARY FIGURE LEGENDS

Figure S1. Identification of SARS-CoV-2-specific T cells through effector function. (**A**) Gating strategy to define CD4$^+$ and CD8$^+$ T cells. Cells were gated on intact, live, singlet CD3$^+$ T cells, followed by further gating for CD4$^+$ or CD8$^+$ T cells. (**B**-**C**) Intracellular cytokine or cytolytic marker expression from CD4$^+$ (***B***) or CD8$^+$ (***C***) T cells, stimulated (*bottom*) or not (*top*) with overlapping 15-mer peptides derived from SARS-CoV-2 spike. Red boxes highlight conditions showing peptide-elicited effector cytokines (IFN-γ, TNF, IL-2, IL-21, CCL4), which were used to define SARS-CoV-2-specific T cells. Cytolytic markers granzyme B, perforin, and CD107a were not specifically induced by peptide stimulation and therefore were not used to define SARS-CoV-2-specific T cells, but were used in subsequent phenotypic analyses.

Figure S2. The frequencies and polyfunctionalities of SARS-CoV-2-specific T cells do not differ between non-pregnant, pregnant, and lactating individuals, but polyfunctional SARS-CoV-2-specific T cell frequencies uniquely associate with IgA responses in those lactating. (**A**-**B**) Percentages of IFN-γ$^+$, TNF$^+$, IL-2$^+$, IL-21$^+$, and CCL4$^+$ cells among total CD4$^+$ (***A***) or CD8$^+$ (***B***) T cells of participants from Study A following SARS-CoV-2 spike peptide stimulation. (**C**-**D**) Percentages of mono- (indicated by the individual cytokines the cells are producing) and poly-functional (Bi$^+$, Tri$^+$ or Quadra$^+$) cytokine-producing cells among SARS-CoV-2-specific CD4$^+$ (***C***) or CD8$^+$ (***D***) T cells from study participants. Bars indicate mean with SD. All comparisons between non-pregnant, pregnant, and lactating groups revealed no significant differences as determined by one-way ANOVA followed by Tukey's multiple comparison's test. For all dot plots, p-values were calculated by one-way ANOVA corrected by Tukey's multiple comparisons tests, Welch's ANOVA corrected by Dunnett T3 multiple comparisons test, or Kruskal-Wallis H tests corrected by Dunn's multiple comparisons test, depending on normality and equality of variance testing.

Data from this figure correspond to that generated from n=10 non-pregnant, n=10 pregnant, and n=18 lactating participants.

Figure S3. Gating strategies to identify classical T cell subsets. $CD4^+$ (***A***) and $CD8^+$ (***B***) T cells were first gated as depicted in Supplemental Figure 1A, and then further gated to identify the following subsets: T_{TM}: $CD45RA^-CD45RO^+CD27^+CCR7^-$, T_{CM}: $CD45RA^-CD45RO^+CD27^+CCR7^+$, T_{EM}: $CD45RA^-CD45RO^+CD27^-CCR7^-$, T_{EMRA}: $CD45RA^+CD45RO^-CCR7^-$, T_{SCM}: $CD45RA^+CD45RO^-CCR7^+CD95^+$, and T_N: $CD45RA^+CD45RO^-CCR7^+CD95^-$. $CD4^+$ T cells were additionally gated for the following two subsets: T_{FH}: $CD45RA^-CD45RO^+PD1^+CXCR5^+$ and T_{reg}: $CD45RA^-CD45RO^+CD25^+CD127^-$. Similar gating strategies were used for both total and SARS-CoV-2-specific T cells. Data shown correspond to total T cells from a representative study participant.

Figure S4. Booster vaccination does not affect the classical subset distribution of SARS-CoV-2-specific T cells in non-pregnant, pregnant, and lactating individuals. (**A**-**C**) Dot plots showing percentages of classical T cell subsets among SARS-CoV-2-specific $CD4^+$ (*left*) or $CD8^+$ (*right*) T cells from non-pregnant (***A***), pregnant (***B***), and lactating (***C***) study participants. Lines delineate paired specimens from the same individuals. The individuals who were lactating for the duration of the study are colored in indicated with purple lines. P-values were calculated by two-sided paired sample *t* tests or Wilcoxon matched-pairs signed rank tests depending on normality and equality of variance testing. Data from this figure correspond to that generated from n=8 non-pregnant, n=7 pregnant, and n=10 lactating participants.

Figure S5. Vaccine-elicited SARS-CoV-2-specific T cells from non-pregnant, pregnant, and lactating individuals display similar expression patterns of CyTOF antigens analyzed in current study. SARS-CoV-2-specific $CD4^+$ (***A***) or $CD8^+$ (***B***) T cells were assessed for expression

levels of all T cell phenotyping markers analyzed by CyTOF. Data are reported as mean signal intensity (MSI), and antigens are listed in the order shown in Supplemental Table S2. Bars indicate mean with SD and dots correspond to individuals. P-values were calculated by one-way ANOVA corrected by Tukey's multiple comparisons test, Welch's ANOVA corrected by Dunnett T3 multiple comparisons test, or Kruskal-Wallis H tests corrected by Dunn's multiple comparisons test, depending on normality and equality of variance testing. Data from this figure correspond to that generated from n=10 non-pregnant, n=10 pregnant, and n=18 lactating participants.

Figure S6. Distribution of FlowSOM clusters of total and SARS-CoV-2-specific T cells do not differ between non-pregnant, pregnant, and lactating individuals. (**A**-**D**) tSNE plots (***A*** and ***C***) and distribution plots (***B*** and ***D***) of FlowSOM clusters of SARS-CoV-2-specific (***A*** and ***B***) or total (***C*** and ***D***) $CD4^+$ or $CD8^+$ T cells among non-pregnant, pregnant, and lactating individuals in Study A. No significant differences were observed in cluster distribution between any of the participant groups, whether assessed by raw p-values (calculated by one-way ANOVA corrected by Tukey's multiple comparisons tests or Kruskal-Wallis H test corrected by Dunn's multiple comparisons tests, depending on normality and equality of variance testing), or after correction using the Benjamini-Hochberg FDR method. Data from this figure correspond to that generated from n=10 non-pregnant, n=10 pregnant, and n=18 lactating participants.

Figure S7. Booster vaccination alters homing properties of vaccine-elicited SARS-CoV-2-specific T cells in a manner affected by pregnancy status. (**A**) Percentages of $CXCR4^+CCR6^+$ cells and $CXCR4^+CD29^+$ cells among SARS-CoV-2-specific $CD4^+$ T cells in non-pregnant, pregnant, and lactating Study B participants. (**B**) Percentages of $CXCR4^+CCR5^+$ cells and $CXCR4^+CCR6^+$ cells among SARS-CoV-2-specific $CD8^+$ T cells in study participants. (**C**) Percentages of $CXCR4^+CCR6^+$ cells and $CXCR4^+CD29^+$ cells among total $CD4^+$ T cells in non-pregnant, pregnant, and lactating study participants. (**D**) Percentages of $CXCR4^+CCR5^+$ cells and

$CXCR4^+CCR6^+$ cells among total $CD8^+$ T cells in study participants. In all panels, lines delineate paired specimens from the same individuals. The individuals who were lactating for the duration of the study are colored in indicated with purple lines. P-values were calculated by two-sided paired sample t tests or Wilcoxon matched-pairs signed rank tests depending on normality and equality of variance testing. Data from this figure correspond to that generated from n=8 non-pregnant, n=7 pregnant, and n=10 lactating participants.

Figure S8. The effects of booster vaccination on the distribution of FlowSOM clusters of SARS-CoV-2-specific T cells from non-pregnant, pregnant, and lactating individuals. (**A**-**B**) Distribution of FlowSOM clusters of SARS-CoV-2-specific $CD4^+$ (***A***) or $CD8^+$ (***B***) T cells among non-pregnant, pregnant, and lactating individuals in Study B. Raw p-values were calculated using two-sided paired sample t tests or Wilcoxon matched-pairs signed rank tests depending on normality and equality of variance testing. Of note, none of the raw p-values remained significant after multiple correction using the Benjamini-Hochberg correction method. (**C**) Cluster A5 of $CD4^+$ T cells highly expresses lymph node homing receptors CCR7 and CD62L and proliferation marker Ki67, while lowly expressing the tissue-homing receptors CXCR4, CCR5, CCR6, and CD29. (**D**) Cluster B1 of $CD8^+$ T cells are stem-like as demonstrated by high expression of CD127 and low expression of terminal differentiation marker CD57, and low levels of checkpoint molecules TIGIT and CTLA4. (**E**) Cluster B4 of $CD8^+$ T cells expresses high levels of cytolytic effectors granzyme B and perforin, and activation markers CD38 and ICOS. (**F**) Cluster B6 of $CD8^+$ T cells expresses high levels of effector cytokine TNFα and cytolytic/degranulation markers granzyme B and CD107a. Significance in panels *C-F* were calculated by two-sided paired sample *t* tests or Wilcoxon matched-pairs signed rank tests depending on normality and equality of variance testing. Data from this figure correspond to that generated from n=8 non-pregnant, n=7 pregnant, and n=10 lactating participants.

Figure S9. Total T cells from non-pregnant, pregnant, and lactating individuals display similar expression patterns of CyTOF antigens analyzed in current study. Total CD4$^+$ (***A***) or CD8$^+$ (***B***) T cells were assessed for expression levels of all T cell phenotyping markers analyzed by CyTOF. Data are reported as mean signal intensity (MSI), and antigens are listed the order shown in Supplemental Table 2. Bars indicate mean with SD and dots correspond to individuals. P-values were calculated by one-way ANOVA corrected by Tukey's multiple comparisons test, Welch's ANOVA corrected by Dunnett T3 multiple comparisons test, or Kruskal-Wallis H tests corrected by Dunn's multiple comparisons test, depending on normality and equality of variance testing. Data from this figure correspond to that generated from n=10 non-pregnant, n=10 pregnant, and n=18 lactating participants.

Figure S10. NK, NKT-like, and γδ T cells frequencies are similar between non-pregnant, pregnant, and lactating individuals, but their expression of HLA-DR trends lower in those lactating. (**A**) Gating strategy to define NK cell subsets. Live, singlet intact cells were gated for CD3$^-$CD4$^-$CD14$^-$ cells, and then gated for CD56 and CD16 expression as indicated. (**B**) Gating strategy to define NKT-like and γδ T cells. Live, singlet intact cells were gated for CD3$^+$ T cells, followed by sub-gating on NKT-like cells (CD56$^+$) and γδ T cells (TCR γδ$^+$) as indicated. (**C**) Percentages of NK, NKT-like, and γδ T cells cell subsets in non-pregnant, pregnant, and lactating individuals. n.s.: non-significant as determined by one-way ANOVA followed by Tukey's multiple comparisons test. (**D**) Mean signal intensity (MSI) of expression level of activation marker HLA-DR among NK, NKT-like, and γδ T cells from study participants. Bars indicate mean with SD, and dots represent individuals. The individuals who were lactating for the duration of the study are indicated in open red circles. P-values were calculated by one-way ANOVA corrected by Tukey's multiple comparisons test or Kruskal-Wallis H tests corrected by Dunn's multiple comparisons test, depending on normality and equality of variance testing. Data from this figure correspond to that generated from n=10 non-pregnant, n=10 pregnant, and n=18 lactating participants.

Figure S11. Breakthrough infection does not affect the magnitude, polyfunctionality, and classical subset distribution of SARS-CoV-2-specific T cells from pregnant and lactating individuals. (**A**) Dot plots showing percentages of SARS-CoV-2-specific $CD4^+$ or $CD8^+$ T cells among total $CD4^+$ or $CD8^+$ T cells from Study C participants. The individuals who were lactating for the duration of the study are indicated with open circles colored brown (vaccination group) and purple (breakthrough infection group). (**B**) SPICE analysis showing frequencies of polyfunctional (colored) or monofunctional (shades of grey) SARS-CoV-2-specific $CD4^+$ (top) and $CD8^+$ (bottom) T cells from study participants. *$p < 0.05$, n.s.: non-significant as determined by Permutation test. (**C**) tSNE dot plots depicting SARS-CoV-2-specific $CD4^+$ and $CD8^+$ T cells from pregnant and lactating study participants. (**D**-**E**) Distributions of classical T cell subsets among SARS-CoV-2-specific $CD4^+$ (***D***) or $CD8^+$ (***E***) T cells from pregnant and lactating study participants. For all bar graphs, bars indicate mean with SD. P-values were calculated by Mann-Whitney U tests or two-sided students' *t* tests, depending on normality and equality of variance testing. Data from this figure correspond to that generated from n=10 vaccine and n=10 breakthrough participants in the pregnant group, and n=18 vaccine and n=7 breakthrough participants in the lactating group.

Figure S12. Distribution of FlowSOM clusters of total and SARS-CoV-2-specific T cells from vaccinated pregnant and lactating individuals that did or did not experience breakthrough SARS-CoV-2 infection. (**A**-**B**) Distribution of FlowSOM clusters of SARS-CoV-2-specific (***A***) or total (***B***) $CD4^+$ or $CD8^+$ T cells among pregnant and lactating individuals in Study C. Raw p-values were calculated using two-sided unpaired sample *t* tests or Mann-Whitney U tests, depending on normality and equality of variance testing. Of note, no p-values were significant after multiple correction using the Benjamini-Hochberg correction method. (**C**) Cluster B6 of SARS-CoV-2-specific $CD8^+$ T cells highly expresses the cytolytic markers granzyme B and CD107a, and lowly expresses the lymph node homing receptors CD62L and CCR7. P-values were calculated by two-

sided student *t* tests or Mann-Whitney U tests depending on normality and equality of variance testing. Data from this figure correspond to that generated from n=10 vaccine and n=10 breakthrough participants in the pregnant group, and n=18 vaccine and n=7 breakthrough participants in the lactating group.

Figure S13. Breakthrough infection decreases the frequencies of γδ T cells in pregnant individuals. Dot plots showing frequencies of NK cells, γδ T cells, and NKT-like cells in pregnant and lactating participants in Study C. Frequencies of $CD56^{++}$ NK cells, $CD56^{+}$ NK cells, and $CD56^{-}$ $CD16^{+}$ NK cells (***A***) are reported as percentages of total NK cells, while frequencies of γδ T cells and NKT-like cells (***B***) are reported as percentages of total $CD3^{+}$ T cells. The individuals who were lactating for the duration of the study are indicated with open circles colored brown (vaccination group) and purple (breakthrough infection group). P-values were calculated by two-sided students' *t* tests or Mann-Whitney U tests, depending on normality and equality of variance testing. Data from this figure correspond to that generated from n=10 vaccine and n=10 breakthrough participants in the pregnant group, and n=18 vaccine and n=7 breakthrough participants in the lactating group.

SUPPLEMENTAL TABLES

Supplemental Table 1. Summary of study participant characteristics

Category	Vaccine[6]	Breakthrough[7]
Participants (N, all female at birth)	38	17
Age[1]	37 (35.25, 40)	36 (32.5, 37)
Gestational age at exposure[2]	21.4 (9.3, 27.5)	20 (11.7, 29.3)
Gestational age at delivery[3]	39 (38, 40)	39 (38, 40)
Race		
White or Caucasian	22 (57.9%)	10 (58.8%)
Asian	5 (13.2%)	3 (17.6%)
Black	0	1 (5.9%)
Other[4]	1 (2.6%)	2 (11.8%)
N/A[5]	10 (26.3%)	0
Ethnicity		
Hispanic or Latino	0	1 (5.9%)
Vaccine		
Pfizer	24 (63.2%)	9 (53%)
Moderna	14 (36.8)	8 (47%)
Status at exposure		
Non-pregnant	10 (26.3%)	0
Lactating	18 (47.4%)	7 (41.2%)
Pregnant	10 (26.3%)	10 (58.8%)

Age[1]: Median years with quantiles
Gestational age[2,3]: Median weeks with quantiles
Other[4]: Middle Eastern, European, or more than one race
N/A[5]: Data not available
Vaccine[6]: Participants that had only received 3 doses of COVID-19 vaccination at the time of sampling
Breakthrough[7]: Participants that had received 3 doses of COVID-19 vaccination followed by a breakthrough SARS-CoV-2 infection

Supplemental Table 2. CyTOF antibodies

Antibody	Clone	Catalog	Metal	Manufacturer
CD196/CCR6	11A9	3141014A	141Pr	Standard BioTools
IL-21*	3A3-N2	513009	142Nd	BioLegend
CD38	HIT2	303535	143Nd	BioLegend
CD195/CCR5	NP6G4	3144007A	144Nd	Standard BioTools
CD16	3G8	3145008B	145Nd	Standard BioTools
CD8	RPAT8	3146001B	146Nd	Standard BioTools
CXCR4	12G5	306523	147Sm	BioLegend
CD278/ICOS	C398.4A	3148019B	148Nd	Standard BioTools
CD25	2A3	3149010B	149Sm	Standard BioTools
CCL4*	D211351	3150004B	150Nd	Standard BioTools
CD107a/LAMP1**	H4A3	3151002B	151Eu	Standard BioTools
TCR γ/δ	11F2	3152008B	152Sm	Standard BioTools
CD62L/L-selectin	DREG56	3153004B	153Eu	Standard BioTools
CD95	50825	MAB326100	154Sm	R&D
CD279/PD-1	EH12.2H7	3155009B	155Gd	Standard BioTools
CD29	TS2/16	3156007B	156Gd	Standard BioTools
CTLA4*	14D3	5012919	157Gd	eBioscience
CD134/Ox40	ACT35	3158012B	158Gd	Standard BioTools
CD197/CCR7	G043H7	3159003A	159Tb	Standard BioTools
CD28	CD28.2	3160003B	160Gd	Standard BioTools
Ki-67*	B56	3161007B	161Dy	Standard BioTools
CD69	FN50	3162001B	162Dy	Standard BioTools
TNF*	Mab11	502941	163Dy	BioLegend
CD45RO	UCHL1	3164007B	164Dy	Standard BioTools
CD127/IL7Rα	A019D5	3165008B	165Ho	Standard BioTools
IL-2*	MQ117H12	3166002B	166Er	Standard BioTools
CD27	L128	3167006B	167Er	Standard BioTools
IFN-γ*	B27	3168005B	168Er	Standard BioTools
CD45RA	HI100	3169008B	169Tm	Standard BioTools
CD3	UCHT1	3170001B	170Er	Standard BioTools
CD185/CXCR5	RF8B2	3171014B	171Yb	Standard BioTools
CD57	HCD57	3172009B	172Yb	Standard BioTools
Granzyme B*	GB11	3173006B	173Yb	Standard BioTools
CD4	SK3	3174004B	174Yb	Standard BioTools
Perforin*	BD48	3175004B	175Lu	Standard BioTools
CD56	NCAM16.2	3176008B	176Yb	Standard BioTools
TIGIT	MBSA43	3209013B	209Bi	Standard BioTools
HLA-DR	TU36	Q22158	Qdot (112Cd)	Invitrogen

*: Intracellular staining
**: Added during peptide stimulation

Figure S1

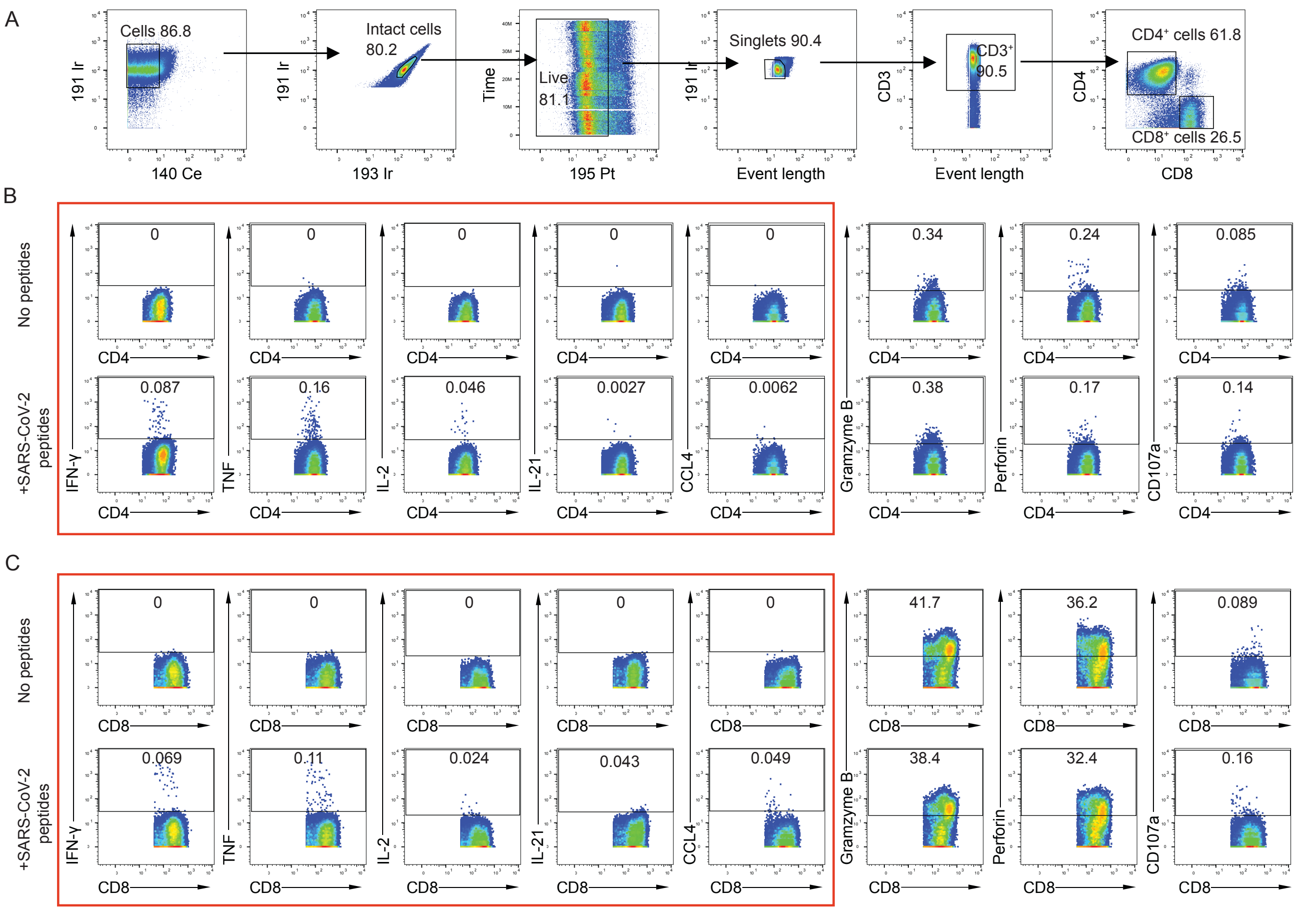
A
Cells 86.8
191 Ir
140 Ce
Intact cells 80.2
193 Ir
Live 81.1
Time
195 Pt
Singlets 90.4
Event length
CD3+ 90.5
CD3
CD4+ cells 61.8
CD8+ cells 26.5
CD4
CD8
B
No peptides
+SARS-CoV-2 peptides
IFN-γ
TNF
IL-2
IL-21
CCL4
Gramzyme B
Perforin
CD107a
0
0
0
0
0
0.34
0.24
0.085
0.087
0.16
0.046
0.0027
0.0062
0.38
0.17
0.14
C
0
0
0
0
0
41.7
36.2
0.089
0.069
0.11
0.024
0.043
0.049
38.4
32.4
0.16

Figure S2

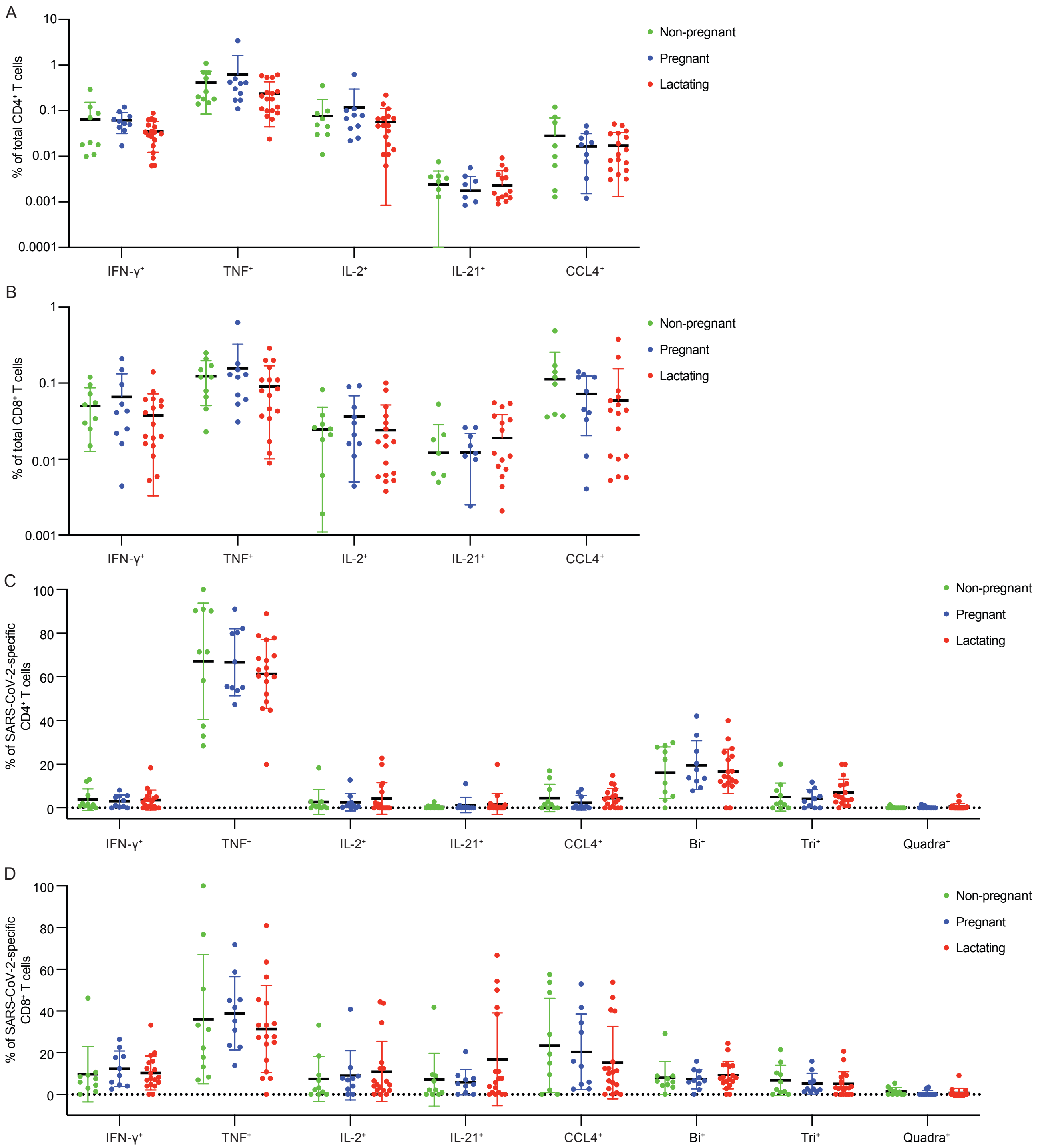
A
10
1
0.1
0.01
0.001
0.0001
% of total CD4+ T cells
IFN-γ+
TNF+
IL-2+
IL-21+
CCL4+
Non-pregnant
Pregnant
Lactating
B
1
0.1
0.01
0.001
% of total CD8+ T cells
IFN-γ+
TNF+
IL-2+
IL-21+
CCL4+
Non-pregnant
Pregnant
Lactating
C
100
80
60
40
20
0
% of SARS-CoV-2-specific CD4+ T cells
IFN-γ+
TNF+
IL-2+
IL-21+
CCL4+
Bi+
Tri+
Quadra+
Non-pregnant
Pregnant
Lactating
D
100
80
60
40
20
0
% of SARS-CoV-2-specific CD8+ T cells
IFN-γ+
TNF+
IL-2+
IL-21+
CCL4+
Bi+
Tri+
Quadra+
Non-pregnant
Pregnant
Lactating

Figure S3

A

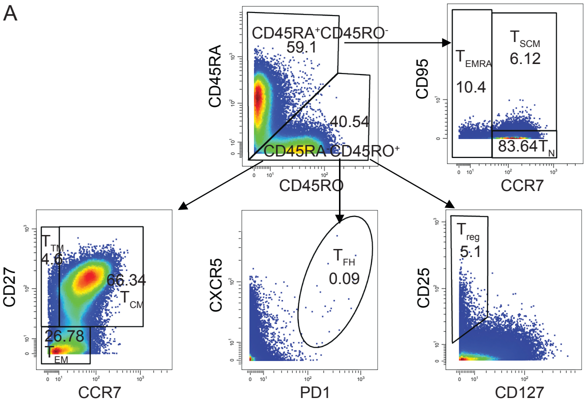
CD45RA
CD45RA+CD45RO-
59.1
40.54
CD45RA-CD45RO+
CD45RO
CD95
TEMRA
10.4
TSCM
6.12
83.64 TN
CCR7
CD27
TTM
4.6
66.34
TCM
26.78
TEM
CCR7
CXCR5
TFH
0.09
PD1
CD25
Treg
5.1
CD127

B

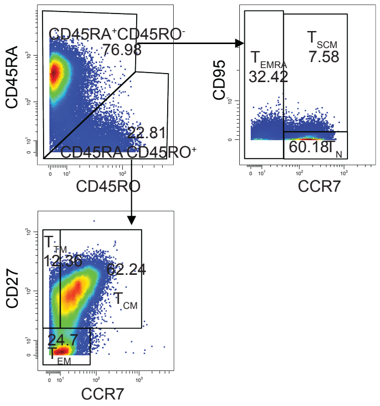
CD45RA
CD45RA+CD45RO-
76.98
22.81
CD45RA-CD45RO+
CD45RO
CD95
TEMRA
32.42
TSCM
7.58
60.18 TN
CCR7
CD27
TTM
12.36
62.24
TCM
24.7
TEM
CCR7

Figure S4

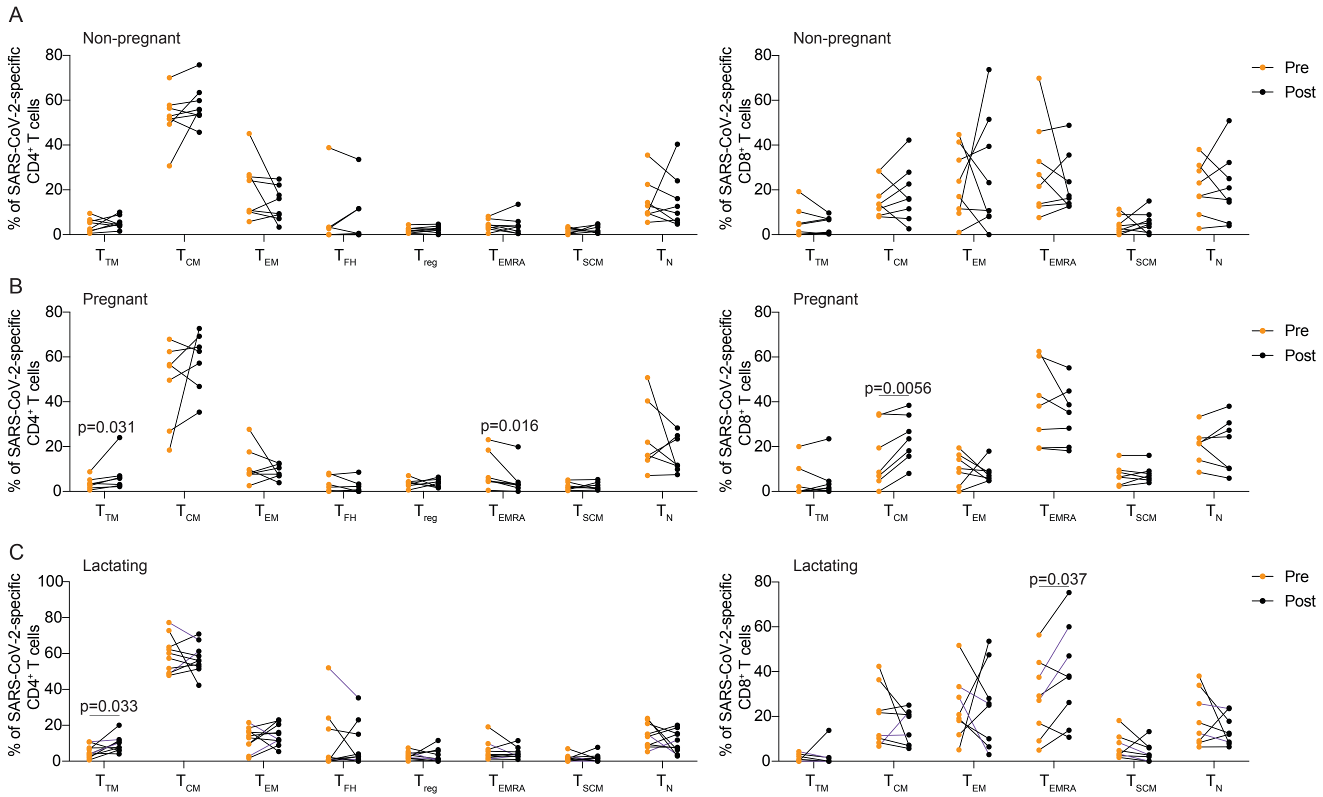

A
Non-pregnant
% of SARS-CoV-2-specific CD4+ T cells
80
60
40
20
0
TTM
TCM
TEM
TFH
Treg
TEMRA
TSCM
TN
Non-pregnant
% of SARS-CoV-2-specific CD8+ T cells
Pre
Post
B
Pregnant
p=0.031
p=0.016
p=0.0056
C
Lactating
100
p=0.033
p=0.037

Figure S5

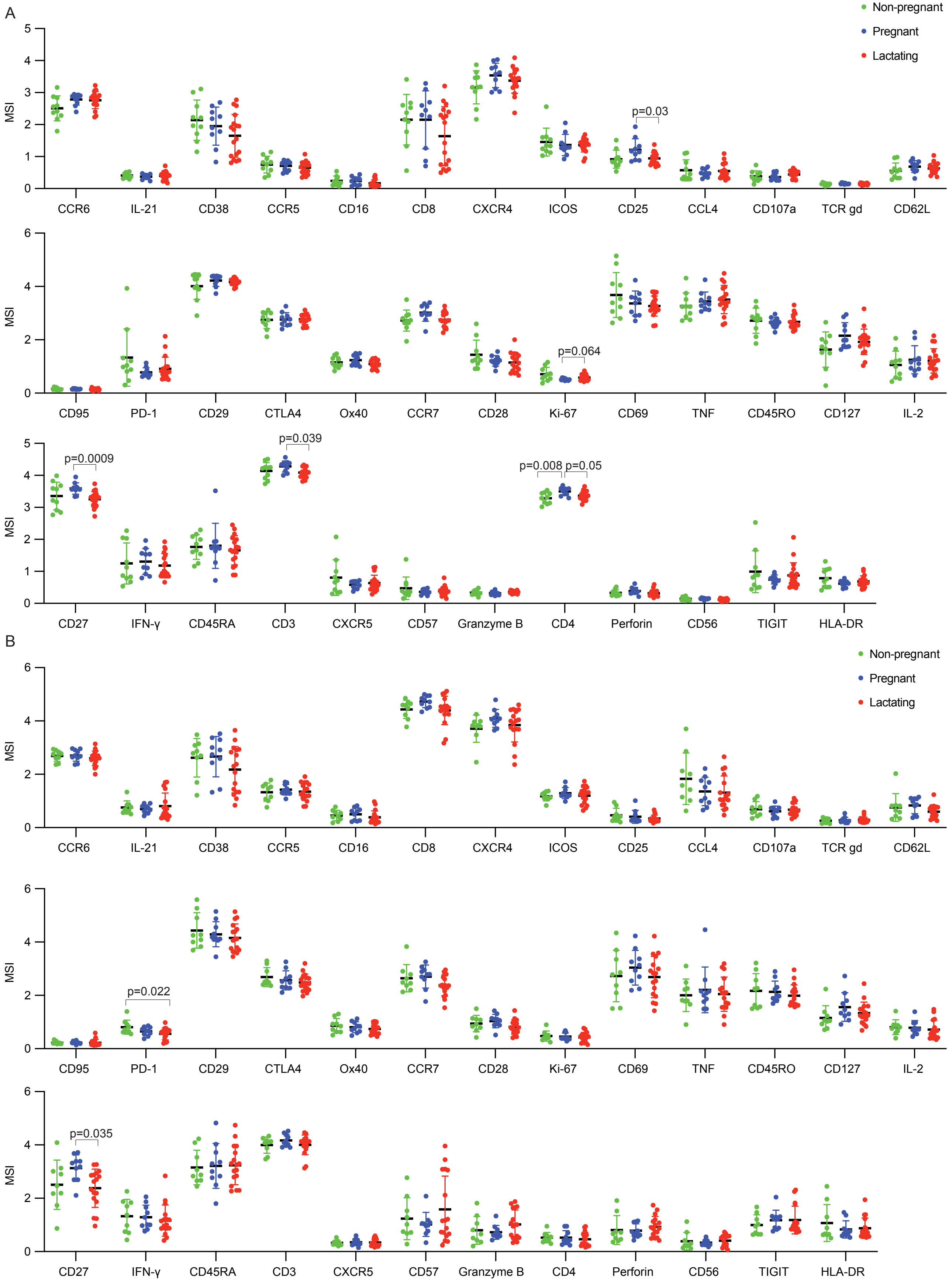
A
Non-pregnant
Pregnant
Lactating
MSI
CCR6
IL-21
CD38
CCR5
CD16
CD8
CXCR4
ICOS
CD25
CCL4
CD107a
TCR gd
CD62L
p=0.03
CD95
PD-1
CD29
CTLA4
Ox40
CCR7
CD28
Ki-67
CD69
TNF
CD45RO
CD127
IL-2
p=0.064
CD27
IFN-γ
CD45RA
CD3
CXCR5
CD57
Granzyme B
CD4
Perforin
CD56
TIGIT
HLA-DR
p=0.0009
p=0.039
p=0.008
p=0.05
B
p=0.022
p=0.035

Figure S6

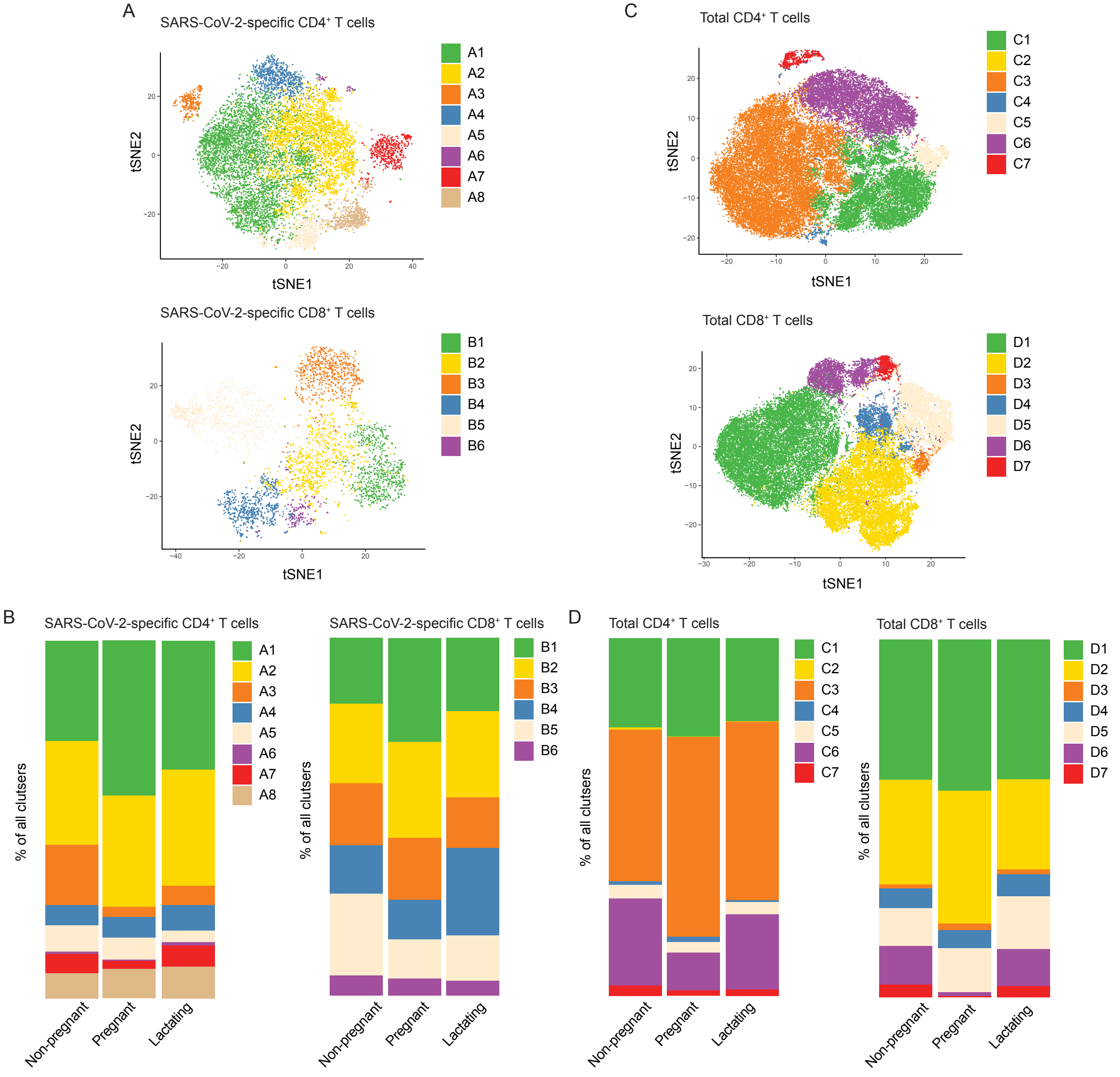
A
SARS-CoV-2-specific CD4⁺ T cells
tSNE2
tSNE1
A1
A2
A3
A4
A5
A6
A7
A8
SARS-CoV-2-specific CD8⁺ T cells
tSNE2
tSNE1
B1
B2
B3
B4
B5
B6
C
Total CD4⁺ T cells
tSNE2
tSNE1
C1
C2
C3
C4
C5
C6
C7
Total CD8⁺ T cells
tSNE2
tSNE1
D1
D2
D3
D4
D5
D6
D7
B
SARS-CoV-2-specific CD4⁺ T cells
% of all clutsers
Non-pregnant
Pregnant
Lactating
SARS-CoV-2-specific CD8⁺ T cells
% of all clutsers
Non-pregnant
Pregnant
Lactating
D
Total CD4⁺ T cells
% of all clutsers
Non-pregnant
Pregnant
Lactating
Total CD8⁺ T cells
% of all clutsers
Non-pregnant
Pregnant
Lactating

Figure S7

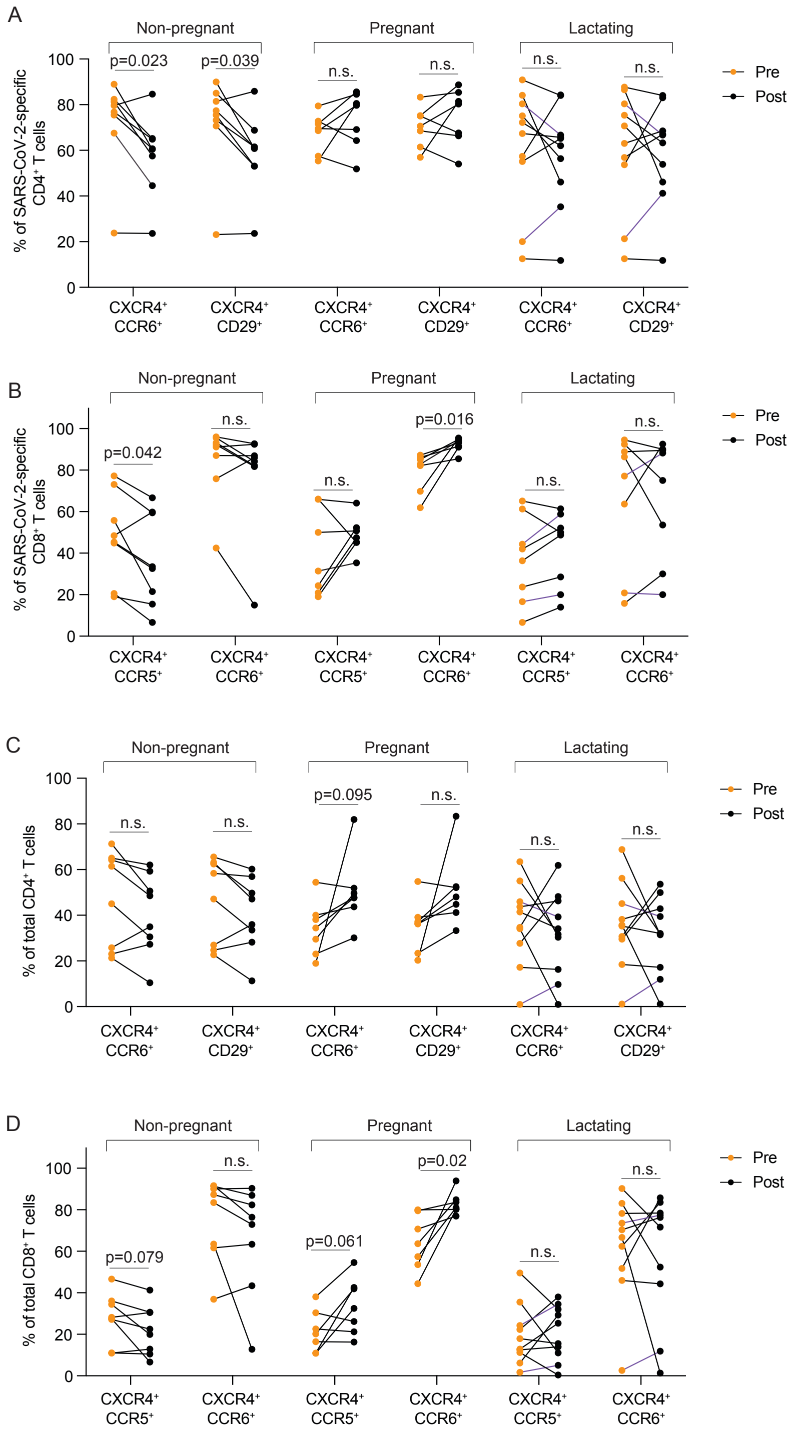
A
Non-pregnant
Pregnant
Lactating
p=0.023
p=0.039
n.s.
% of SARS-CoV-2-specific CD4+ T cells
CXCR4+ CCR6+
CXCR4+ CD29+
Pre
Post
B
p=0.042
p=0.016
% of SARS-CoV-2-specific CD8+ T cells
CXCR4+ CCR5+
CXCR4+ CCR6+
C
p=0.095
% of total CD4+ T cells
D
p=0.079
p=0.061
p=0.02
% of total CD8+ T cells

Figure S8

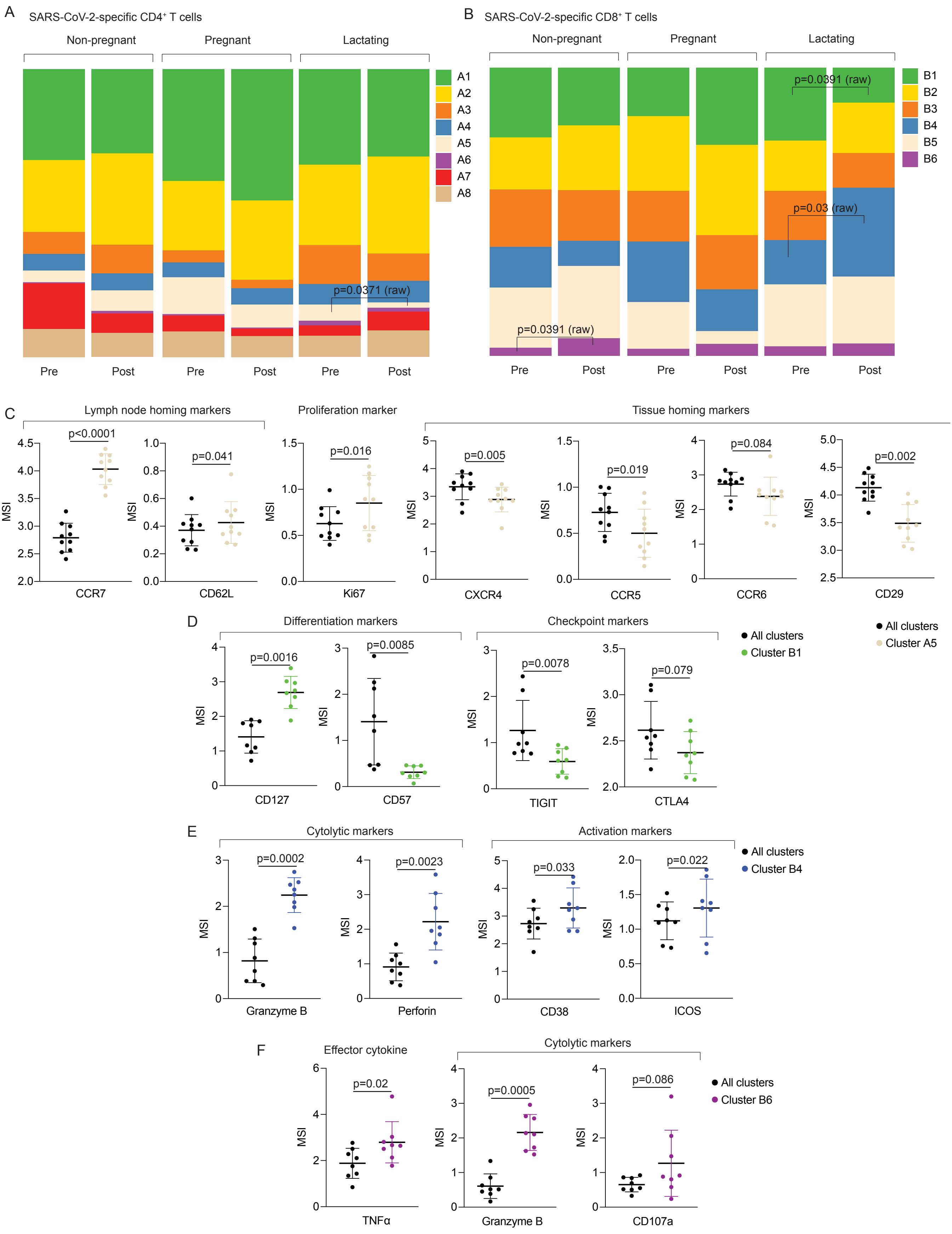
A
SARS-CoV-2-specific CD4⁺ T cells
Non-pregnant
Pregnant
Lactating
A1
A2
A3
A4
A5
A6
A7
A8
p=0.0371 (raw)
Pre
Post
Pre
Post
Pre
Post
B
SARS-CoV-2-specific CD8⁺ T cells
Non-pregnant
Pregnant
Lactating
B1
B2
B3
B4
B5
B6
p=0.0391 (raw)
p=0.03 (raw)
p=0.0391 (raw)
Pre
Post
Pre
Post
Pre
Post
C
Lymph node homing markers
Proliferation marker
Tissue homing markers
MSI
p<0.0001
CCR7
p=0.041
CD62L
p=0.016
Ki67
p=0.005
CXCR4
p=0.019
CCR5
p=0.084
CCR6
p=0.002
CD29
All clusters
Cluster A5
D
Differentiation markers
Checkpoint markers
p=0.0016
CD127
p=0.0085
CD57
p=0.0078
TIGIT
p=0.079
CTLA4
All clusters
Cluster B1
E
Cytolytic markers
Activation markers
p=0.0002
Granzyme B
p=0.0023
Perforin
p=0.033
CD38
p=0.022
ICOS
All clusters
Cluster B4
F
Effector cytokine
Cytolytic markers
p=0.02
TNFα
p=0.0005
Granzyme B
p=0.086
CD107a
All clusters
Cluster B6

Figure S9

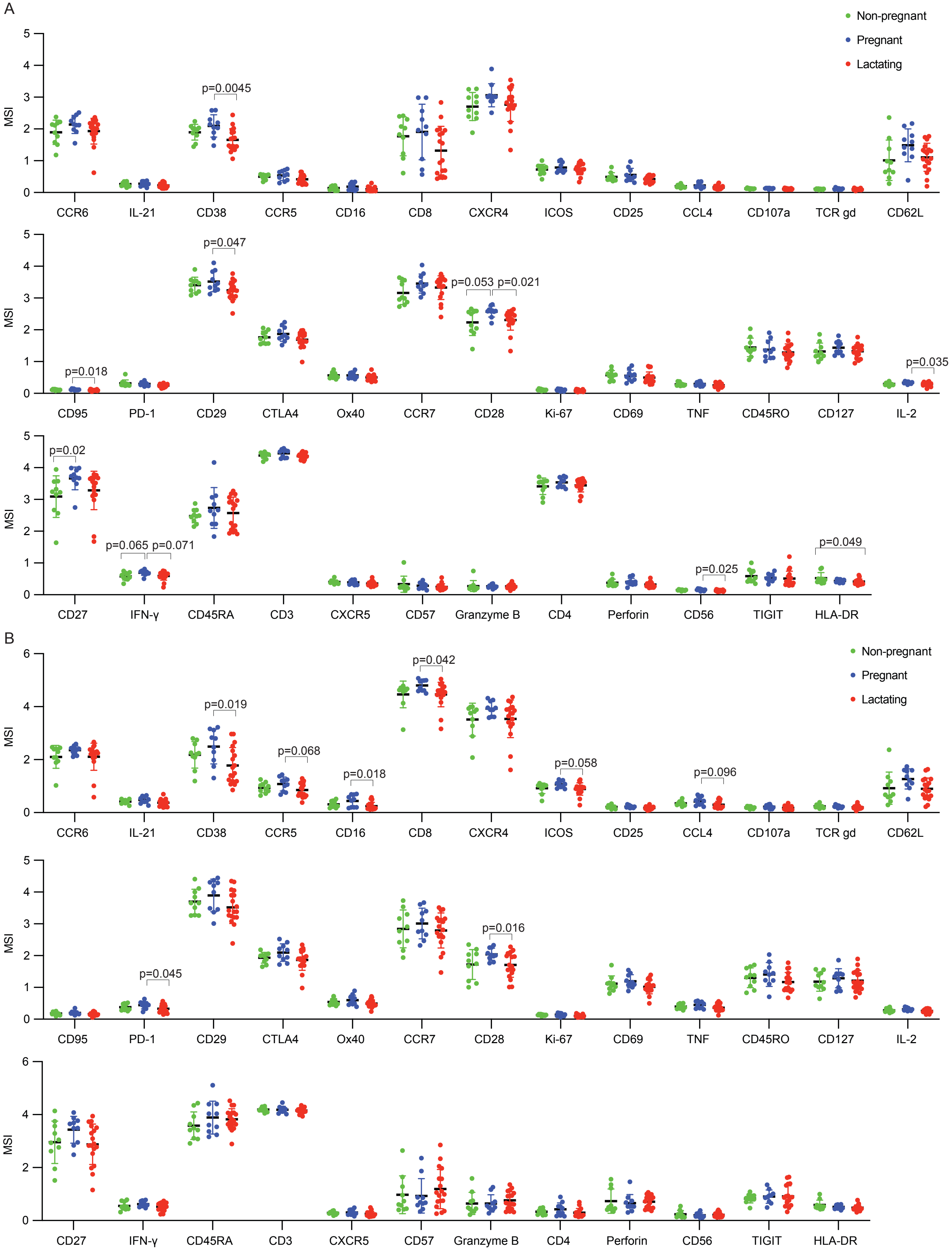
A
Non-pregnant
Pregnant
Lactating
MSI
5
4
3
2
1
0
p=0.0045
CCR6
IL-21
CD38
CCR5
CD16
CD8
CXCR4
ICOS
CD25
CCL4
CD107a
TCR gd
CD62L
p=0.047
p=0.053
p=0.021
p=0.018
p=0.035
CD95
PD-1
CD29
CTLA4
Ox40
CCR7
CD28
Ki-67
CD69
TNF
CD45RO
CD127
IL-2
p=0.02
p=0.065
p=0.071
p=0.025
p=0.049
CD27
IFN-γ
CD45RA
CD3
CXCR5
CD57
Granzyme B
CD4
Perforin
CD56
TIGIT
HLA-DR
B
6
p=0.042
p=0.019
p=0.068
p=0.018
p=0.058
p=0.096
p=0.016
p=0.045

Figure S10

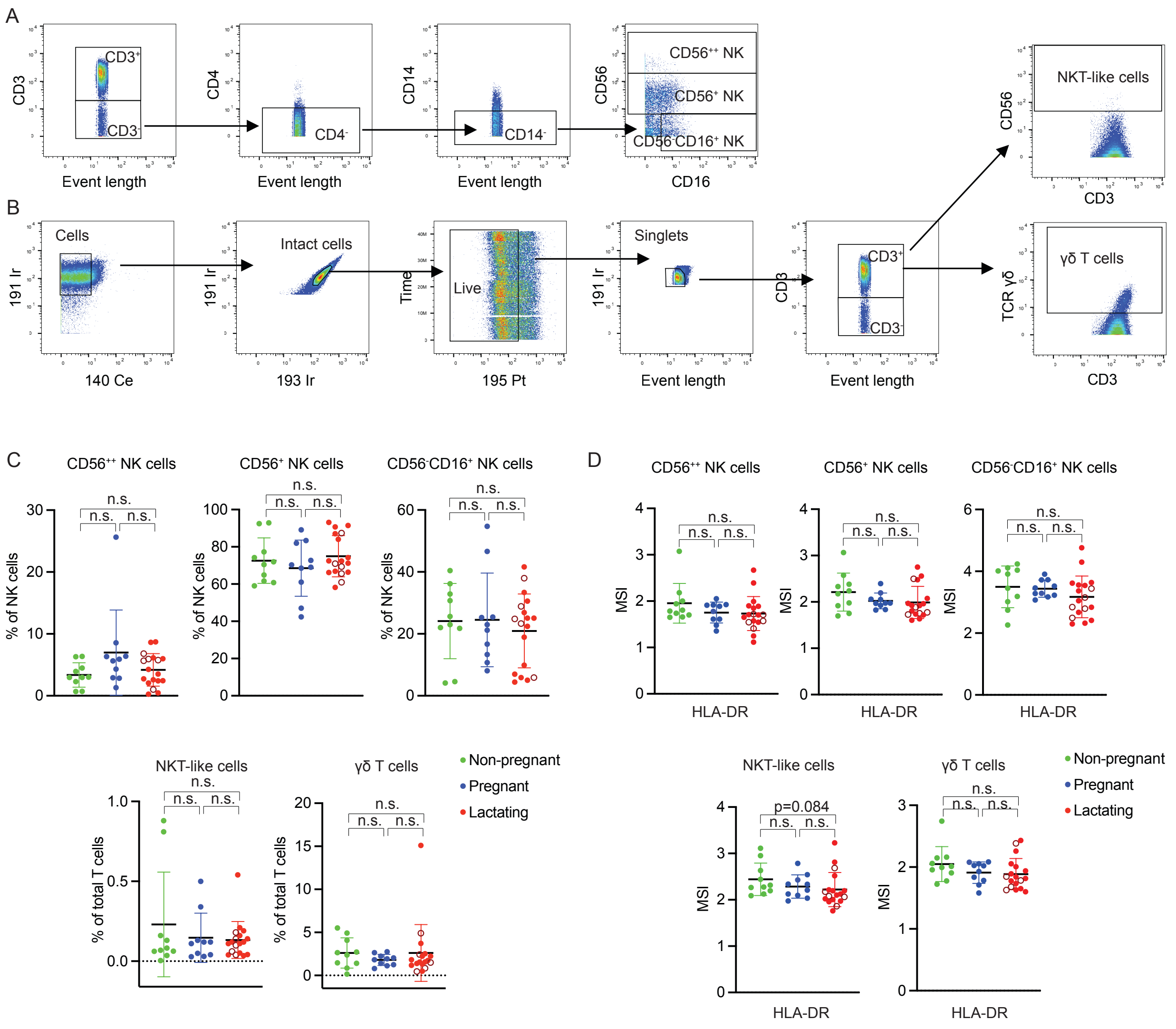
A
CD3
CD3+
CD3-
Event length
CD4
CD4-
Event length
CD14
CD14-
Event length
CD56
CD56++ NK
CD56+ NK
CD56-CD16+ NK
CD16
CD56
NKT-like cells
CD3
B
Cells
191 Ir
140 Ce
Intact cells
191 Ir
193 Ir
Time
Live
195 Pt
Singlets
191 Ir
Event length
CD3
CD3+
CD3-
Event length
TCR γδ
γδ T cells
CD3
C
CD56++ NK cells
CD56+ NK cells
CD56-CD16+ NK cells
% of NK cells
n.s.
NKT-like cells
γδ T cells
% of total T cells
Non-pregnant
Pregnant
Lactating
D
CD56++ NK cells
CD56+ NK cells
CD56-CD16+ NK cells
MSI
HLA-DR
n.s.
NKT-like cells
γδ T cells
p=0.084
Non-pregnant
Pregnant
Lactating

Figure S11

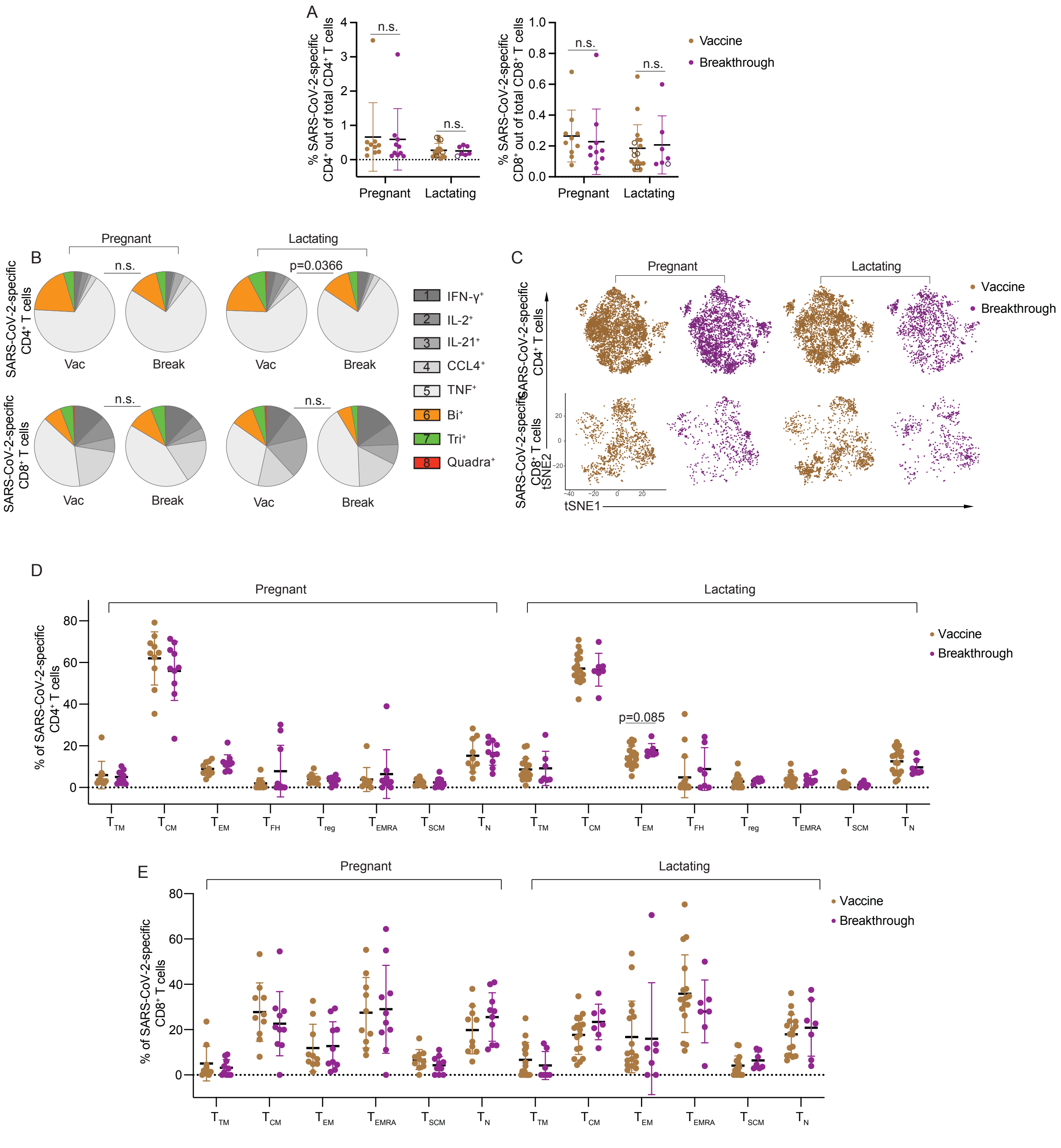
A
% SARS-CoV-2-specific CD4+ out of total CD4+ T cells
% SARS-CoV-2-specific CD8+ out of total CD8+ T cells
n.s.
Pregnant
Lactating
Vaccine
Breakthrough
B
Pregnant
Lactating
n.s.
p=0.0366
SARS-CoV-2-specific CD4+ T cells
SARS-CoV-2-specific CD8+ T cells
Vac
Break
1 IFN-γ+
2 IL-2+
3 IL-21+
4 CCL4+
5 TNF+
6 Bi+
7 Tri+
8 Quadra+
C
tSNE1
tSNE2
D
% of SARS-CoV-2-specific CD4+ T cells
p=0.085
TTM
TCM
TEM
TFH
Treg
TEMRA
TSCM
TN
E
% of SARS-CoV-2-specific CD8+ T cells

Figure S12

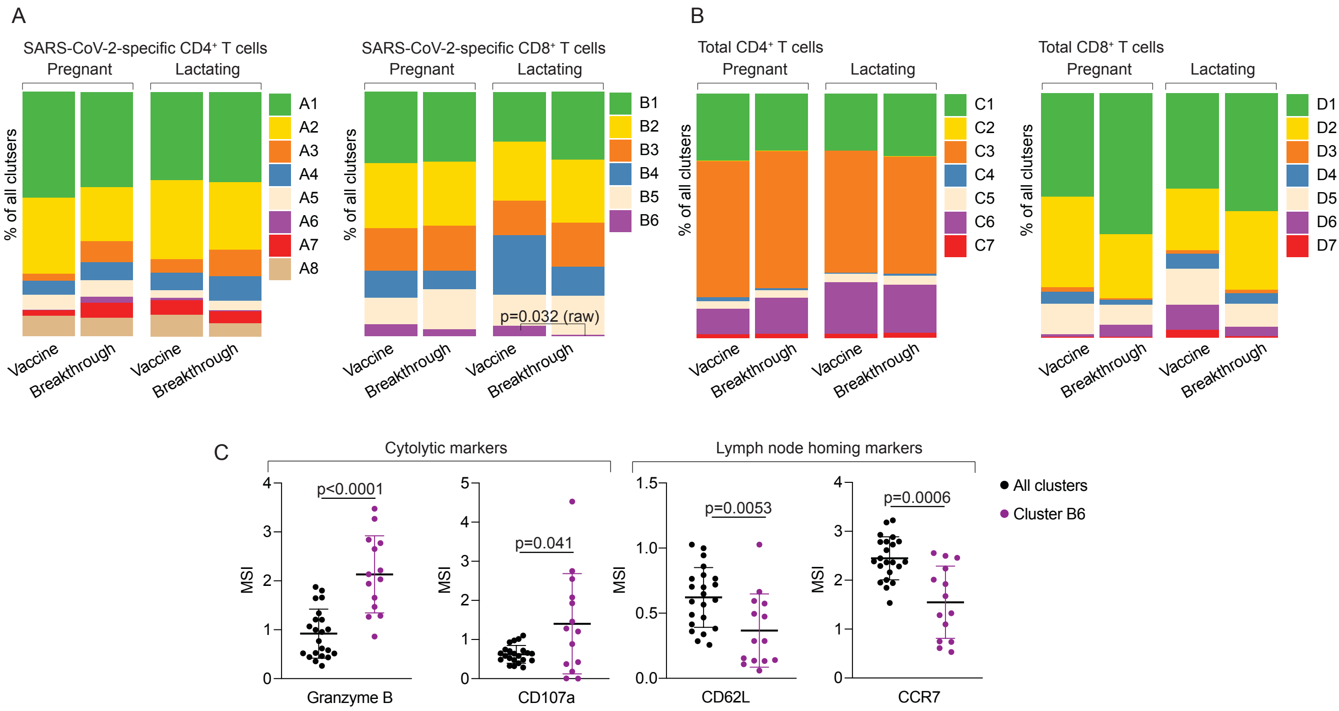
A
SARS-CoV-2-specific CD4+ T cells
Pregnant
Lactating
% of all clutsers
Vaccine
Breakthrough
A1
A2
A3
A4
A5
A6
A7
A8
SARS-CoV-2-specific CD8+ T cells
B1
B2
B3
B4
B5
B6
p=0.032 (raw)
B
Total CD4+ T cells
C1
C2
C3
C4
C5
C6
C7
Total CD8+ T cells
D1
D2
D3
D4
D5
D6
D7
C
Cytolytic markers
Lymph node homing markers
MSI
p<0.0001
p=0.041
p=0.0053
p=0.0006
Granzyme B
CD107a
CD62L
CCR7
All clusters
Cluster B6

Figure S13

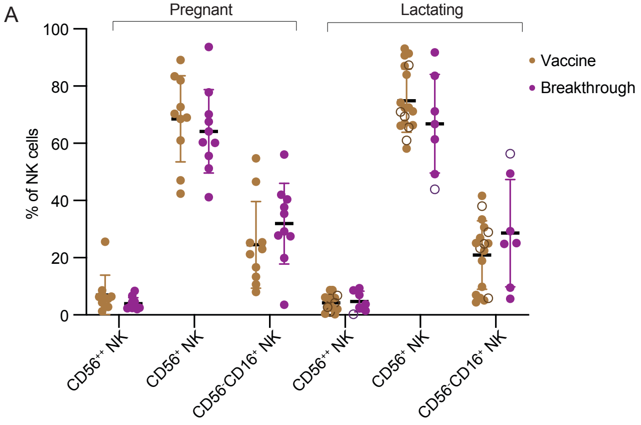

A
Pregnant
Lactating
100
80
60
40
20
0
% of NK cells
Vaccine
Breakthrough
CD56++ NK
CD56+ NK
CD56-CD16+ NK
CD56++ NK
CD56+ NK
CD56-CD16+ NK

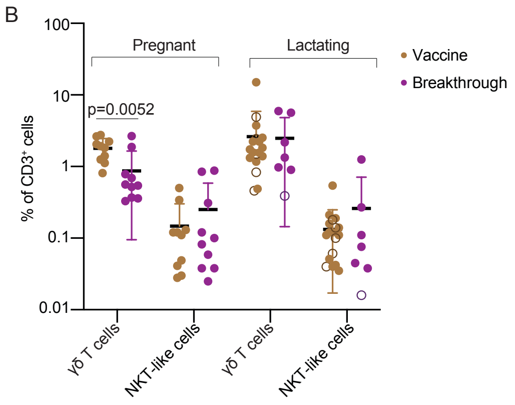

B
100
10
1
0.1
0.01
% of CD3+ cells
Pregnant
Lactating
p=0.0052
Vaccine
Breakthrough
γδ T cells
NKT-like cells
γδ T cells
NKT-like cells

SUPPLEMENTARY METHODS

CyTOF data analyses

Data normalization and beads removal

Unprocessed flow cytometry standard (fcs) files were first normalized using CyTOF software (Standard BioTools, version 6.7). The median dual count values of the metal isotopes on EQ four element calibration beads were used as the internal standard for data normalization. Data files were then manually gated on 191Ir^{+}140Ce^{-} to remove beads from the analysis.

Batch normalization

A total of 320 fcs files in total were generated for this study. This was comprised of 80 samples (from 55 participants), each of which were comprised of both CD4^{+} and CD8^{+} T cell datasets, which in turn included samples stimulated or not with SARS-CoV-2 spike peptides. Specimens stained within the same processing run were considered a batch. Specimens run at each batch were deliberately not matched by immunity (breakthrough infection or vaccination) or physiological states (non-pregnant, pregnant, lactating), to randomize the order of data acquisition. An aliquot of anchor samples (cisplatin-stained PBMCs) was run each time, and was used for batch normalization. Batch normalization was performed as previously described by implementing the BatchAdjust function (1).

Identification of cells of interest

Sequential gating was used to identify intact, live, and singlet cells (Supplemental Figure 1A) followed by sub-gating on subsets of interest (Supplemental Figure 3, Supplemental Figure 10, A and B). Of note, natural killer (NK) cells were gated upon using methods similar to those previously described (2): intact, live, singlet, CD3^{-}CD4^{-}CD14^{-} cells were gated into CD56^{++}, CD56^{+}, and CD56^{-}CD16^{+} NK cells were gated based on CD56 vs CD16 expression as indicated

in Supplemental Figure 10A. Natural killer T cells (Supplemental Figure S10) were identified as $CD3^+CD56^+$ cells, but were referred to as NKT-like cells since not all $CD3^+CD56^+$ cells are *bona fide* CD1d-restricted NKT cells (3). γδ T cells were identified as $CD3^+$ T cells expressing the γδ TCR (Supplemental Figure 10B).

Identification of SARS-CoV-2-specific T cells

SARS-CoV-2-specific T cells were identified based on specific expression of intracellular cytokines or cytolytic markers after peptide stimulation. Cytokines included the canonical Th1 cytokine IFN-γ, the inflammatory cytokines TNF and CCL4, the proliferative cytokine IL-2 commonly produced by $CD4^+$ T cells, and the T_{FH}-associated cytokine IL-21. Cytolytic markers included the serine protease granzyme B and the pore-forming protein perforin secreted by cytotoxic cells, as well as the degranulation marker CD107a. Both effector cytokines (IFN-γ, TNF, IL-2, IL-21, CCL4) and cytolytic markers (Granzyme B, Perforin, CD107a) were assessed for the ability to specifically identify antigen-specific T cells by comparing the peptide-stimulated to the non peptide treated counterpart. The following rules were adopted, similar to methods reported (4), to establish which cytokines or cytolytic markers could be used to specifically define SARS-CoV-2-specific T cells: (1) positive counts in unstimulated sample were less than 5 events, or the frequency of positive counts among total $CD4^+$ or $CD8^+$ T cells was lower than 0.1%; (2) positive counts in the peptide-stimulated sample were not less than 5, or the frequency of positive counts was higher than 0.1%; (3) the difference between frequency of unstimulated and frequency of peptide-stimulated sample cells was not less than 0.01%; (4) the ratio between frequency of peptide-stimulated and frequency of unstimulated sample cells was greater than 5. Effectors that met all 4 criteria were IFN-γ, TNF, IL-2, IL-21, and CCL4, for both SARS-CoV-2-specific $CD4^+$ and $CD8^+$ T cells (Supplemental Figure 1, B and C). For phenotypic analysis, we excluded samples from donors who had less than 5 SARS-CoV-2-specific T cells. These only occurred among SARS-CoV-2-specific $CD8^+$ T cells, and such donors were non-

pregnant participant C01018, pregnant participant V11166, and lactating participants V11168 and V11067.

tSNE

Arcsinh data was transformed in R (version 4.2.3) and the Rtsne (version 0.16) or RColorBrewer (version 1.1-3) packages were used for data visualization. The parameters were set as follows: down sampling *n* = 1,000 for baseline T cells and *n* = 8,000 for SARS-CoV-2-specific T cells, iteration = 1,000, perplexity = 30, and theta = 0.5. For data representation, we implemented packages including ggplot2 (version 3.4.4) and reshape 2 (version 1.4.4).

Cell polyfunctionality analyses

Cell polyfunctionality analyses were performed using the SPICE algorithm (SPICE, version 6.1) as previously described (5) with some modifications. SARS-CoV-2-specific T cells defined by Boolean gating were selected and percentages of single cytokine positive cells among total $CD4^+$ or $CD8^+$ T cells were calculated (FlowJo, version 10.10.0) as an input dataset for polyfunctionality analyses, serving as the variable “value”. $CD4^+$ or $CD8^+$ T cells were set as the mutually exclusive variable “group”. Vaccination status was set as the variable “overlay”. Each individual cytokine was set as the variable “category”. Iterations of permutation tests were set as 10,000 and the highlight value was set as 0.05.

FlowSOM analysis

FlowSOM (version 2.12.0) clustering was performed in R (version 4.2.3), following loading of the packages ggplot2, reshape2, gridEXtra, cowplot, dplyr, and RColorBrewer. Meta-cluster number determination was established based on expression patterns of lineage markers CD45RA, CD45RO, CD27, and CCR7, as well as lineage-defining effector cytokines (IFN-γ, TNF, IL-2, IL-21, and CCL4). This resulted in identification of 8 clusters of SARS-CoV-2-specific $CD4^+$ T cells,

6 clusters of SARS-CoV-2-specific $CD8^+$ T cells, 7 clusters of total $CD4^+$ T cells, and 7 clusters of total $CD8^+$ T cells. The number of subclusters was set to the default setting of 40. All remaining parameters were kept as default.

REFERENCES

1. Schuyler RP, Jackson C, Garcia-Perez JE, Baxter RM, Ogolla S, Rochford R, et al. Minimizing Batch Effects in Mass Cytometry Data. *Front Immunol.* 2019;10:2367.
2. Zhao NQ, Vendrame E, Ferreira AM, Seiler C, Ranganath T, Alary M, et al. Natural killer cell phenotype is altered in HIV-exposed seronegative women. *PLoS One.* 2020;15(9):e0238347.
3. Krijgsman D, de Vries NL, Skovbo A, Andersen MN, Swets M, Bastiaannet E, et al. Characterization of circulating T-, NK-, and NKT cell subsets in patients with colorectal cancer: the peripheral blood immune cell profile. *Cancer Immunol Immunother.* 2019;68(6):1011-24.
4. Yin K, Peluso MJ, Luo X, Thomas R, Shin MG, Neidleman J, et al. Long COVID manifests with T cell dysregulation, inflammation and an uncoordinated adaptive immune response to SARS-CoV-2. *Nat Immunol.* 2024;25(2):218-25.
5. Roederer M, Nozzi JL, and Nason MC. SPICE: exploration and analysis of post-cytometric complex multivariate datasets. *Cytometry A.* 2011;79(2):167-74.
